# SputOMICs identifies common and distinct markers in cystic fibrosis and chronic obstructive pulmonary disease

**DOI:** 10.1038/s41598-025-32565-y

**Published:** 2025-12-24

**Authors:** Dario L. Frey, Barbara Helm, Matteo Guerra, Matthias Hagner, Junyan Lu, A. Susanne Dittrich, Sabine Wege, Ralf Eberhardt, Felix J. F. Herth, Olaf Sommerburg, Carsten Schultz, Alexander H. Dalpke, Ursula Klingmüller, Marcus A. Mall, Sébastien Boutin

**Affiliations:** 1 Department of Translational Pulmonology, University of Heidelberg, Heidelberg, Germany; 2 Translational Lung Research Center (TLRC), German Center for Lung Research (DZL), Heidelberg, Germany; 3 Division of Systems Biology of Signal Transduction, German Cancer Research Center (DKFZ), Heidelberg, Germany; 4 Molecular Medicine Partnership Unit (MMPU), European Molecular Biology Laboratory, Heidelberg (EMBL), Germany; 5 Institute for Computational Biomedicine, University Hospital Heidelberg, Heidelberg, Germany; 6 Department of Pulmonology and Critical Care Medicine, Thoraxklinik at the University of Heidelberg, Heidelberg, Germany; 7 Pneumology & Critical Care Medicine, Asklepios Klinik Barmbek, Hamburg, Germany; 8 Division of Pediatric Pulmonology & Allergology and Cystic Fibrosis Center, Department of Pediatrics, University of Heidelberg, Heidelberg, Germany; 9 Department of Chemical Physiology and Biochemistry, Oregon Health & Science University, Portland, OR USA; 10 Department of Infectious Diseases, Medical Microbiology and Hygiene, Heidelberg University, Medical Faculty Heidelberg,, Heidelberg, Germany; 11 Department of Pediatric Respiratory Medicine, Immunology and Critical Care Medicine and Cystic Fibrosis Center, Charité - Universitätsmedizin Berlin, corporate member of Freie Universität Berlin and Humboldt-Universität Zu Berlin, Berlin, Germany; 12 German Center for Child and Adolescent Health (DZKJ), Partner Site Berlin, Berlin, Germany; 13 German Center for Lung Research (DZL), Associated Partner Site Berlin, Berlin, Germany; 14 Cluster of Excellence ImmunoPreCept, Charité - Universitätsmedizin Berlin, Berlin, Germany; 15 Institute of Medical Microbiology, University of Lübeck and University Hospital Schleswig-Holstein, Campus Lübeck, Lübeck, Germany; 16 Airway Research Center North (ARCN), German Center for Lung Research (DZL), Großhansdorf, Germany; 17 Present Address: Proteomics Core Facility, German Cancer Research Center (DKFZ), Heidelberg, Germany; 18 Present Address: Department of Biochemical and Cellular Pharmacology, Genentech, South San Francisco, CA USA

**Keywords:** Cystic fibrosis, COPD, Biomarkers, Multi-omics, Proteomics, Microbiome, Biomarkers, Computational biology and bioinformatics, Diseases, Medical research

## Abstract

Cystic fibrosis (CF) and chronic obstructive pulmonary disease (COPD) are muco-obstructive lung diseases. Knowledge of molecular processes has much improved therapeutic options in CF, whereas much less is known for COPD, a disease affecting an increasing number of patients. Here, we report a multilayer workflow integrating microbiome, inflammation and proteome profiling with clinical data to identify disease specific characteristics in sputum. Our proof-of-concept study shows that CF sputum is dominated by *Pseudomonas* and *Staphylococcus*, exhibits heightened neutrophilic inflammation, and a severe protease-antiprotease imbalance. In contrast, COPD displays heterogeneous microbiome composition, eosinophilic inflammation, and altered extracellular matrix remodeling. Proteome-based cellular deconvolution identifies disease-specific immune cell signatures, underscoring the complexity, especially in COPD. Multi-omics factor analysis suggests that matrisome and nucleotide metabolism changes may act as disease discriminators, though future confirmation in larger cohorts is needed. These findings highlight the potential of our integrated approach to uncover sputum biomarkers as tools for patient stratification and personalized therapeutic strategies in CF and COPD.

**Supplementary Information:**

The online version contains supplementary material available at 10.1038/s41598-025-32565-y.

## Introduction

Cystic fibrosis (CF) and chronic obstructive pulmonary disease (COPD) are chronic muco-obstructive lung diseases characterized by chronic neutrophilic airway inflammation and dysbiosis, leading to a protease-antiprotease imbalance and progressive structural lung damage^1–3^. Despite these shared pathological features, CF and COPD differ significantly in their underlying causes, clinical presentations, and therapeutic approaches. However, their overlapping features, in particular chronic neutrophilic airway inflammation, mucus obstruction, and airway dysbiosis, contribute to a shared clinical phenotype of muco-obstructive disease. A direct comparison of these two diseases enables the establishment and evaluation of an integrative pipeline for the identification of both common and disease-specific biomarkers and pathways that may guide future efforts in personalized diagnosis and treatment, especially in the more heterogeneous COPD population.

CF is an autosomal recessive disorder caused by mutations in the cystic fibrosis transmembrane conductance regulator (CFTR) gene, which results in dysfunctional CFTR channels^2,4,5^. This defect causes abnormal mucus properties and impaired mucociliary clearance, fostering chronic airway infection, inflammation, and structural damage, collectively contributing to a gradual decline in lung function^1,2^. In contrast, COPD is primarily an acquired disease caused by long-term exposure to harmful particles and gases, particularly tobacco smoke, but also occupational dust and air pollution^6^. Affecting over 391 million people globally, COPD is projected to become the leading cause of death worldwide within the next 15 years^6^.

Therapeutic advancements for CF have been transformative in recent years, particularly with the development of CFTR modulators such as Elexacaftor/Tezacaftor/Ivacaftor^7–10^. These therapies target the underlying molecular defect, resulting in substantially improved clinical outcomes for CF patients^7,8^. In addition to improving pulmonary health, CFTR modulators have been shown to partially normalize the sputum proteome and increase microbiome diversity by reducing pathogenic dominance of classical pathogen such as *Pseudomonas aeruginosa*^11,12^. On the contrary, progress in targeted therapies for COPD has lagged^3^. The diagnosis of COPD remains to be based on lung function measurements using forced spirometry to determine the forced expiratory volume in 1 s (FEV_1_)^13^. As specified by the Global Initiative for Chronic Obstructive Lung Disease (GOLD), COPD patients are subcategorized based on the FEV_1_ into stages ranging from mild (GOLD I) to very severe (GOLD IV)^14^. Moreover, COPD is frequently associated with severe comorbidities, including cardiovascular disease and metabolic syndrome, which complicate disease management and treatment strategies^15^. Although chronic inflammation and airway microbiota alterations in COPD are increasingly recognized as critical contributors to disease progression, research has predominantly focused on plasma and serum biomarkers^16–19^. Sputum, a readily accessible and non-invasive sample, has been studied in advanced COPD^20–23^, but its full potential remains underexplored, particularly for integrated multi-omics analyses that can reveal cross-omic molecular patterns and airway-specific changes beyond isolated biomarker studies.

Sputum analysis offers a unique and accessible view of changes in soluble factors, immune cells and the microbiome of the lower respiratory tract. It thus provides insights into alterations underlying chronic respiratory diseases such as CF and COPD. Unlike invasive methods, such as tissue biopsies or blood analyses, sputum collection is a non-invasive, patient-friendly approach that enables direct assessment of airway inflammation, immune cell profiles, protease activity, and microbiome dynamics. Lately, in CF, proteomic studies of sputum samples allowed us to identify proteomic changes during modulator therapy and to compare those to healthy individuals^11,24^. In COPD, much research has focused on comparing stable patients with exacerbated patients based on the analysis of plasma or serum samples that primarily provide insights into systemic changes e.g. in inflammatory factors and immune cells^16,17,21^. Up to now, studies of sputum samples from COPD patients have focused on individual levels such as eosinophil counts^20^, microbiome^22^, mucin levels^23^, and metabolomic biomarkers^21^, and in those, the emphasis was mostly on comparing smokers with nonsmokers. While sputum analysis is particularly advantageous for evaluating disease states and therapeutic outcomes over time, current studies have focused mainly on individual parameters and have not yet integrated several layers of analysis nor performed a comparative analysis of both chronic muco-obstructive lung diseases.

CF and COPD involve chronic neutrophilic inflammation, with granule protein release contributing to persistent lung damage^25,26^. However, the inflammatory profiles and clinical implications of these conditions are distinct. In CF, chronic infection and inflammation are closely linked, with therapeutic efforts targeting both aspects^10,11^. In COPD, inflammation, exacerbations, and airway microbiota alterations are interconnected. Still, current treatments often fail to address these links adequately^15,27,28^. This unmet need for anti-inflammatory and anti-infective therapies, particularly those tailored to specific disease characteristics, underscores the importance of comprehensive, integrative approaches to disease characterization.

To address these gaps, we established an integrative SputOMICs workflow that was employed for a detailed comparative analysis of microbiome, inflammation, protease-antiprotease imbalance, and proteome in sputum samples from patients with CF or COPD, as well as from healthy controls in an observational study. Although differentiating cystic fibrosis, chronic obstructive pulmonary disease, and healthy individuals based on age and cell counts is clinically straightforward, this study serves as a proof-of-concept for an integrated multi-omics workflow designed to reveal complex molecular and immune signatures beyond routine clinical parameters. Our multi-omics approach combines microbiome studies with mass spectrometry-based proteomics for direct quantification of protein abundance, revealing disease-specific changes in CF and COPD sputum, particularly proteins associated with adaptive immunity pathways and shifts in protease-antiprotease balance, suggesting a key role for microbiome alterations in both conditions but also provides evidence for distinct disease-associated factors. Integrating proteomic and microbiome insights, our study unravels the mechanistic complexities of CF and COPD. It identifies sputum markers that provide a basis to refine disease classification and pave the way for individualized treatment options. This workflow has potential for application across other respiratory or inflammatory diseases to enhance molecular understanding and guide personalized interventions.

## Material and methods

Additional information is provided in the online supplement.

### Study population

This prospective observational study was approved by the University of Heidelberg’s ethics committee (S-046/2009, S-370/2011, S-041/2018). All patients gave written informed consent and the research has been performed in accordance with the Declaration of Helsinki. Table 1 and Supplementary Table 2 & 4 provide demographics as age and sex and extensive clinical characteristics as BMI, Pancreatic insufficiency status, CFTR genotype and the 6-min walking test as well as several lung specific parameters as FEV_1_% predicted, RV % predicted and Diff % predicted. The healthy control group consisted of volunteers without known chronic respiratory disease.

**Table 1:** Demographics and clinical characteristics of study population.

Subjects	n	CF	COPD	Healthy controls
		38	18	10
Age (years)	Median (range)	29.33 (20.80–73.83)	66.57 (50.70–78.20)	30.68 (27.32–49.04)
Sex	n, females/males	11/27	10/8	5/5
BMI (kg/m^2^)	Median (range)	20.96 (15.79–30–96)	21.96 (18.59–44.46)	-
FEV_1_% predicted*	Median (range)	57.19 (16.93–92.88)	34.10 (18.30–47.70)	-
CFTR genotype				
F508del/F508del	n (%)	14 (36.84)	-	-
F508del/other	n (%)	20 (52.63)	-	-
Other/other	n (%)	4 (10.53)	-	-
Pancreatic insufficiency	n (%)	35 (92.11)	-	-
CFTR therapy				
Lumacaftor/Ivacaftor	n (%)	3 (7.89)	-	-
Tezacaftor/Ivacaftor	n (%)	2 (5.26)	-	-
GOLD stage (3/4)	n (%)	-	12(66.66)/6(33.33)	-
RV % predicted	Median (range)	-	208.50 (170.3–288.1)	-
Diff % predicted	Median (range)	-	34.15 (21.50–74.20)	-
6 min walking test (m)	Median (range)	-	328.50 (152.00–432.0)	-

*FEV_1_% predicted only available from 32 CF patients.

### Microbiome analysis

Samples used for the microbiome analysis were incubated with PMA™ dye (Biotium Inc., Hayward, USA). Subsequently, the copy number of the 16S rDNA gene was quantified, and libraries were prepared as published earlier^29^. The sequence data was processed using Dada2, and amplicon sequence variants (ASVs) were counted and classified using Silva database v138.1.

### Sputum collection and sample pre-treatment for inflammatory biomarker analysis

**Fig. 1:**
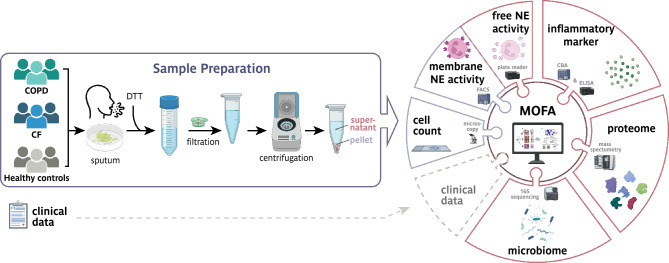
Overview of the SputOMICs workflow. The individual steps of sample processing and analysis as well as integration of clinical data and the multiomics data are schematically represented. Chronic obstructive pulmonary disease (COPD); dithiothreitol (DTT); neutrophil elastase (NE); flow cytometry (FACS); cytometric bead array (CBA); enzyme-linked immunosorbent assay (ELISA); Multi-omics factor analysis (MOFA).

Sputum samples were treated as described previously^30–33^. In brief, spontaneously expectorated sputum from CF and COPD patients and induced sputum of healthy controls stored on ice immediately after production and into three aliquots within two hours. To minimize contamination, sputum samples were visually inspected and carefully separated from saliva prior to further processing following established sputum processing protocols^11,34,35^. The aliquot for the microbiome was processed on the same day as described in detail in the supplement. The aliquot for the other studies was divided into supernatant and cell pellet (Fig. 1). The supernatant was used for examinations of the levels of endogenous anti-proteases (A1AT, A1AT/NE complex, SLPI, TIMP1, and LTB_4_ via ELISA (Abnova, R&D Systems, eBioscience). Inflammatory cytokines (IL-1α, IL-1β, IL-5, IL-6, IL-8, IL-10, TNF-α, TGF-β_1_ and IFN-γ) were quantified using cytokine bead arrays (BD Biosciences). The cell pellet was utilized for the determination of neutrophil elastase (NE) membrane activity and of differential cell counts of May-Grünwald-Giemsa stained cell preparations indicated as percentage of total cell numbers.

### Soluble and surface-associated neutrophil elastase activity

Soluble and surface-associated neutrophil elastase (NE) activity was measured using Förster resonance energy transfer (FRET) based reporters NEmo-1 and NEmo-2 (Sirius Fine Chemicals, Bremen, Germany) as previously described^30,32,33^, applying the recently introduced small molecule FRET flow procedure, to quantify NE activity in the supernatant fraction as well as on the surface of sputum neutrophils in the cell pellet fraction^30,36^.

### Proteomic analysis by mass spectrometry

The protein content of thawed sputum supernatants was determined. Per sample, 5µg of protein was reduced, alkylated, digested, and cleaned up by an Auto-SP3 protocol and analyzed by an Ultimate 3000 HPLC-Orbitrap Exploris 480 mass spectrometer in data-independent mode. Spectronaut 15.6 was used with the UniProt Human-reviewed canonical reference proteome for data analysis.

### Statistical analysis and multi-omics factor analysis

Statistical analyses were performed with R Statistical Software (v4.1.2; R Core Team 2021). Group-wise comparisons used a pairwise Wilcoxon rank sum test, adjusted for multiple comparisons with p-value < 0.05 considered significant. Kmeans clustering based on the Morisita-Horn dissimilarity index was defined as microbiome clusters. Proteomics data was analyzed with MSPypeline^37^, and the proteome, microbiome, and inflammatory marker dataset was integrated using the ‘MOFA2’ package (version 1.60)^38^.

## Results

### The SputOMICs workflow allows an integrative multilevel analysis of a study cohort

To characterize changes in the sputum of CF and COPD patients and to uncover the complex interplay of multiple factors driving the diseases, we established a SputOMICs workflow (Fig. 1) combining diverse cellular and molecular profiles and integrates this multilevel analysis with clinical data. Sputum is a highly viscous fluid. Therefore, to make it accessible to the molecular studies in our workflow, it was homogenized using a 10% solution of Sputolysin (DTT), filtered, and centrifuged to generate a cellular pellet and a supernatant fraction (Fig. 1). The cellular pellet was used for cell typing, determining the number of inflammatory cells, and measuring the activity of membrane-associated Neutrophil Elastase (NE). Alterations in the microbiome, inflammatory factors, soluble NE activity, protease/antiprotease levels, and the proteome were analyzed in the supernatants. A Multi-Omics Factor Analysis (MOFA) was employed for the integrative data analysis to provide systems-wide insights into disease-relevant changes in the sputum. To evaluate our workflow, we performed a proof-of-concept study providing preliminary insights and examined sputum samples from a representative cohort comprising 38 CF patients, 18 COPD patients, and 10 healthy controls. The cohort captured diverse clinical and demographical features of both lung diseases as COPD patients were older (67 vs. 29 years), exhibited slightly higher BMI (22 vs. 21 kg/m^2^), and had a lower FEV_1_ percent predicted (34% vs 57%; GOLD stage III and IV) compared to CF patients (Table 1 and Supp. Fig. 1 A). Spontaneous sputum from CF and COPD patients was used to maintain methodological consistency, while for the healthy controls, induced sputum was collected and separated from saliva.

### Microbiome profiles are divergent in CF and COPD

**Fig. 2:**
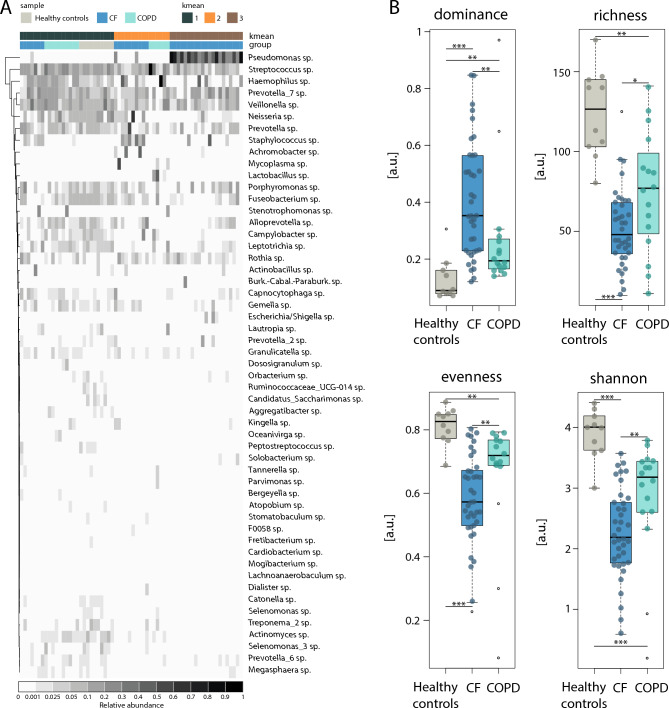
Comparison of microbiomes in sputum from patients with CF, patients with COPD and healthy controls. (**A**) Heatmap of the relative abundance of the top 53 ASVs, healthy controls; CF; COPD are indicated by color. The kmean cluster are indicated in teal, orange and brown. (**B**) Calculated biodiversity parameters: dominance, richness, evenness and Shannon index for each cluster. Healthy controls (●); CF (●); COPD (●), outliers are shown as empty circles (○).

The level of the respiratory microbiome is critical for shaping inflammation and structural changes in the respiratory tract, particularly in chronic respiratory diseases. While the analysis of sputum samples has much advanced our understanding of the role of the microbiome in CF, significantly less is known for COPD. To address this gap, we compared the sputum microbiomes of healthy controls, CF patients, and COPD patients using advanced 16S RNA sequencing (see Supp. Fig. 2 and 3 for quality control of the sequencing). Our analysis identified three distinct microbiome clusters by k-means clustering (Fig. 2A and Supp. Fig. 4). Microbiome cluster 1 encompassed all healthy controls, most COPD patients, and a subset of CF patients, while microbiome cluster 2 included additional COPD and CF patients. Microbiome cluster 3 was exclusively composed of CF samples. CF sputum microbiomes were dominated by well-known CF pathogens, with *Staphylococcus* (prevalent in microbiome clusters 2) and *Pseudomonas* (highly abundant in microbiome clusters 3). In contrast, COPD sputum microbiomes often resembled the structure of healthy controls (microbiome clusters 1) or were characterized by dominance of *Haemophilus*, *Streptococcus*, or *Lactobacillus* (microbiome clusters 2). Principal Coordinates Analysis (PCoA) revealed a distinct separation between the three microbiome groups (Supp. Fig. 5). Biodiversity analysis underscored these differences. Healthy controls exhibited the most diverse microbiomes, characterized by low dominance, high richness and evenness, and the highest Shannon index (Fig. 2B). In contrast, CF sputum displayed the highest dominance and the lowest richness, evenness, and Shannon index (Fig. 2B). COPD microbiomes demonstrated intermediate diversity between the profiles of CF and healthy controls. Despite these differences, microbial copy numbers and biomass were comparable across groups (Supp. Fig. 1D).

These findings underscore distinct microbiome differences between CF and COPD. CF is dominated by pathogenic bacteria like *Pseudomonas* and *Staphylococcus*, which drive inflammation and tissue damage, while COPD exhibits more diverse microbiomes linked to broader pathophysiological processes.

### Inflammatory profiles in CF and COPD sputum are distinct

**Fig. 3:**
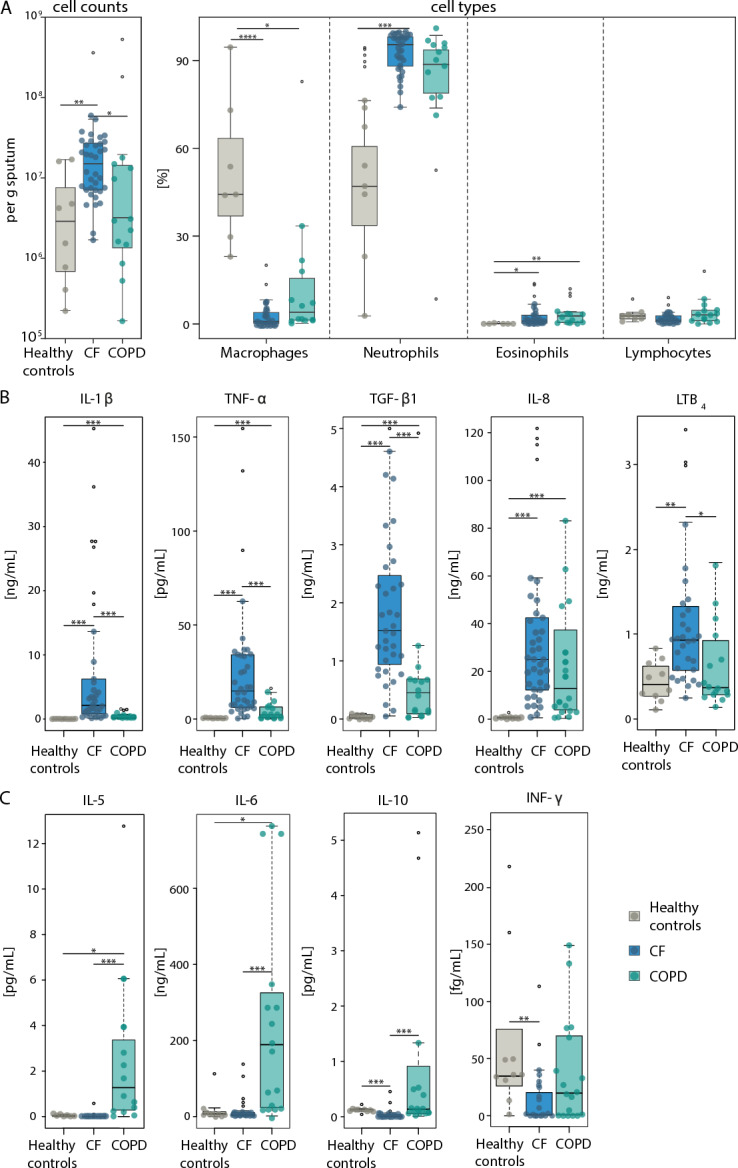
Total and differential inflammatory cell counts and inflammatory markers in sputum from patients with CF, patients with COPD, and healthy controls. Boxplot of (**A**) total inflammatory cells per gram sputum (healthy controls ● n = 8; CF ● n = 36; COPD ● n = 14, and differential cell count showing the percentages macrophages, neutrophils, eosinophils and lymphocytes (Healthy controls ● n = 7; CF ● n = 36; COPD ● n = 14. Boxplots of (**B**) IL-1β, TNF-α, TGF-β_1_, IL-8 and LTB_4_, C IL-5, IL-6, IL-10 and INF-γ are given. Healthy controls ● n = 8–10; CF ● n = 31–36; COPD ● n = 13–18, numbers per graph are given in the Supp. Table 1, outliers are shown as empty circles (**○**).

Based on the analysis of blood and sputum samples, chronic neutrophilic inflammation has been established as a common hallmark of both diseases^25,26^, causing persistent respiratory symptoms and irreversible airflow limitation^39^. While in CF, inflammation is dominated by an overwhelming presence of neutrophils^33^, COPD exhibits a more heterogeneous pattern involving elevated eosinophils, macrophages, and lymphocytes^20,40–43^. To better understand these differences, we analyzed the cellular fraction of our sputum samples (Fig. 3 and Supp. Fig. 1B,C). We observed that total cell numbers were significantly higher in CF and moderately increased in COPD than in healthy individuals (Fig. 3A, left panel). Both diseases exhibited substantial neutrophilia, which was far more pronounced in CF. Interestingly, eosinophil levels were elevated in both diseases, while macrophage numbers were significantly reduced (Fig. 3A, right panel).

Despite these similarities in the cellular composition of the sputum, we found striking differences between the two diseases regarding the presence of inflammatory factors in the sputum supernatants (sample inclusion details are provided in Supp. Tab. 1). CF samples had notably higher levels of key inflammatory markers such as IL-1β, TNF-α, TGF-β_1_, IL-8, and LTB_4_ (Fig. 3B), reflecting the intensity of neutrophilic inflammation in this condition. On the other hand, COPD samples showed elevated levels of IL-5, IL-6, and IL-10 (Fig. 3C).

These differences in inflammatory signatures point to a unique inflammatory environment in COPD.

### Protease-antiprotease balance is dysregulated in CF and COPD sputum

**Fig. 4:**
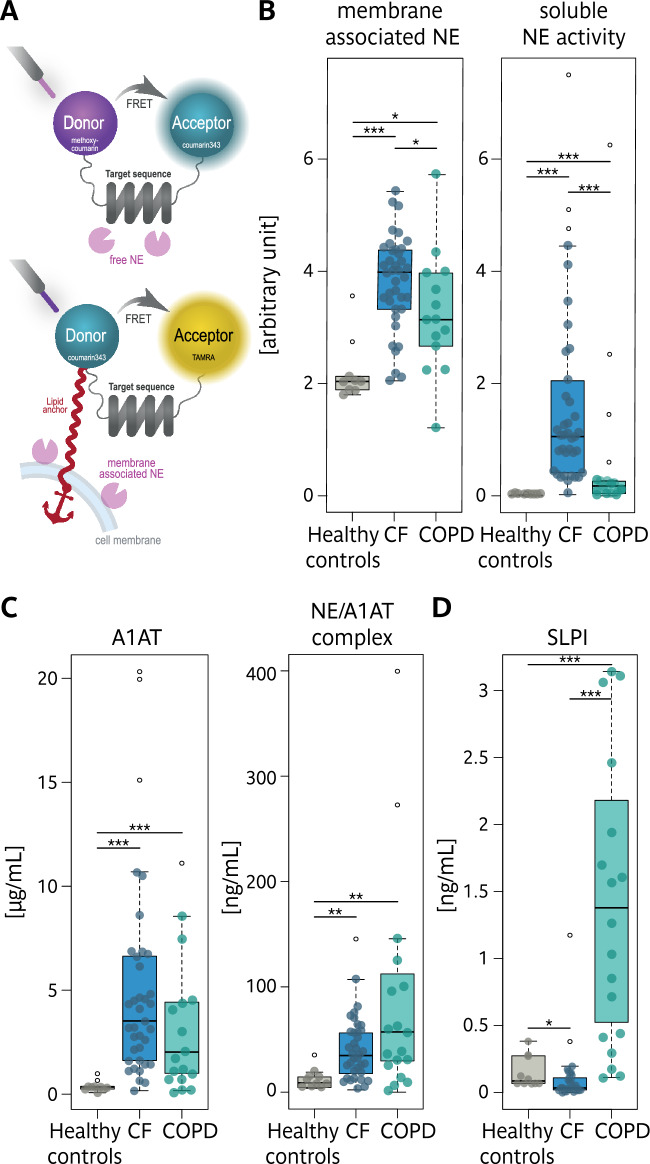
Comparison of membrane-associated and soluble NE activity, as well as levels of endogenous anti-proteases and complexes in sputum from patients with CF, patients with COPD and Healthy controls. (**A**) Principle of FRET reporters NEmo-1 for soluble NE activity and NEmo-2 for quantification of membrane-associated NE activity. Boxplots of (**B**) membrane-associated NE on the surface of sputum neutrophils, and soluble NE activity in sputum supernatant (**C**) A1AT and NE/A1AT complex, and (**D**) SLPI. Healthy controls (●); CF (●); COPD (●), outliers are shown as empty circles (○).

For the integrity of the lung parenchyma, a balance between proteases and antiproteases is critical, and both diseases exhibit dysregulated protease activity, leading to tissue damage and chronic inflammation. However, the underlying pathways and severity of this imbalance could vary considerably between CF and COPD. To address this, we assessed both soluble and membrane-bound neutrophil elastase (NE) activity, as membrane-associated NE has been shown to precede the detection of soluble NE and is less susceptible to endogenous inhibitors^30,44^. Notably, membrane-bound NE activity has been shown to be elevated in mice with CF-like lung disease and in patients with CF even before soluble NE activity can be detected, while the anti-protease shield remains intact in early stages of lung disease^33,45^. Unlike soluble NE, membrane-associated NE is protected from endogenous inhibitors, and elevated membrane-bound NE activity can contribute to structural lung damage even in the absence of elevated soluble NE activity^32,46,47^. To specifically capture this membrane-bound NE activity, we previously developed a flow cytometry-based assay^36^.Analysis of the cellular sputum fraction revealed that membrane-bound NE activity was similarly elevated in both diseases (Fig. 4A,B, right panel; surface markers in Supp. Fig. 1 C) suggesting comparable levels of neutrophil activation. Yet surprisingly, soluble NE activity in sputum supernatants was dramatically higher in CF compared to COPD, indicating a severe breakdown of the anti-protease defence system in CF (Fig. 4A,B, left panel). Further analysis uncovered distinct protease-antiprotease patterns in the two diseases. In COPD, levels of the key inhibitor α−1-antitrypsin (A1AT) were increased, along with the formation of NE/A1AT complexes (Fig. 4C). In contrast, CF sputum showed reduced levels of Secretory Leukocyte Protease Inhibitor (SLPI) (Fig. 4D).

Thus, while CF is characterized by excessive protease activity and insufficient inhibition, COPD shows signs of an adaptive response, with increased inhibitor production to counterpart protease activity.

### Proteomic sputum profiling identifies distinct alterations in CF and high heterogeneity in COPD

**Fig. 5:**
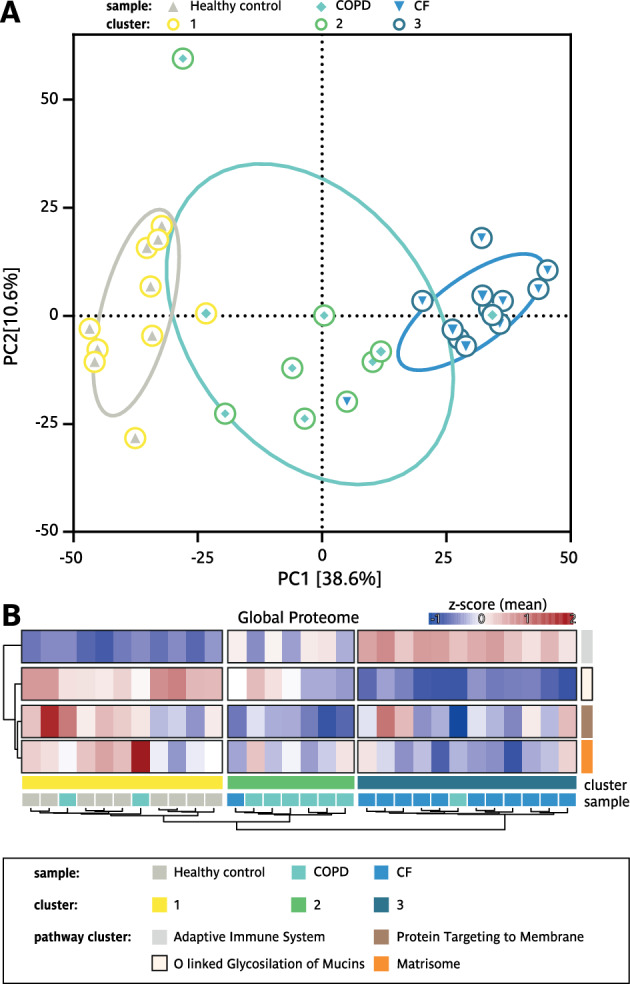
Comparison of sputum proteome of patients with CF, patients with COPD and Healthy controls. (**A**) Principal component analysis (PCA) of all detected proteins, circles indicate 50% of the 95% distribution interval. (**B**) Heatmaps of the global proteome analysis, top mean z-score of the four main protein clusters, and the most enriched pathway per cluster, as well as Healthy controls (●); CF patients (●); COPD patients (●).

**Fig. 6:**
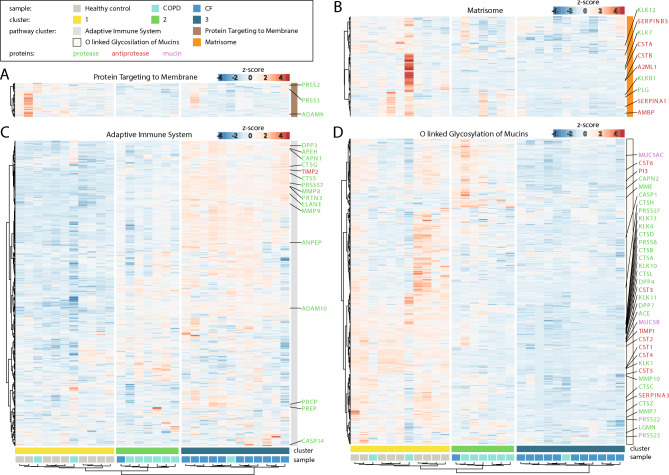
Detailed comparison of sputum proteome of patients with CF, patients with COPD and Healthy controls. Detailed heatmap of the main pathway clusters. All proteins which are proteases are indicated in green, antiproteases in red and mucin related proteins in purple. Sample cluster 1 (●); sample cluster 2 (●); sample cluster (●). Healthy controls (●); COPD (●) and CF (●).

**Fig. 7:**
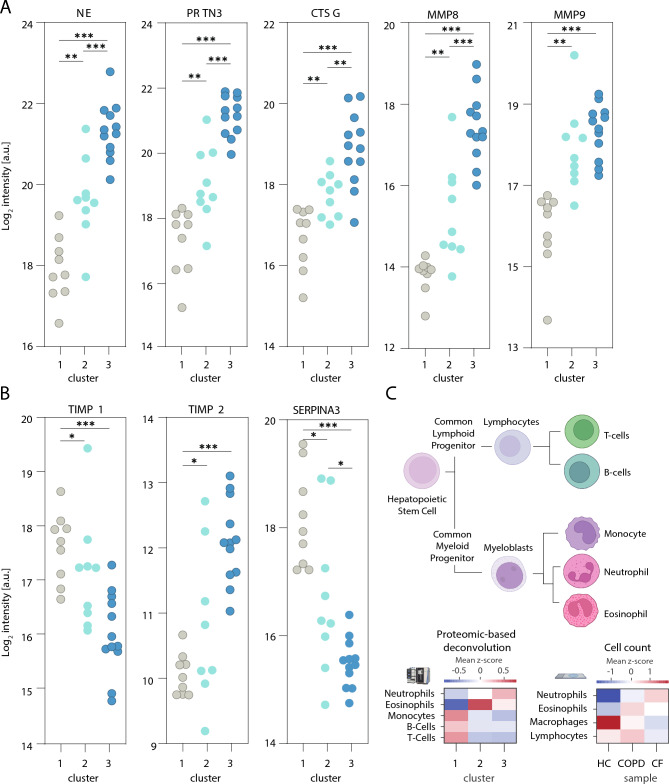
Comparison of proteases, antiproteases and mucins as well as proteomic cell deconvolution from the sputum of patients with CF, patients with COPD and Healthy controls. (**A**) Confetti plots displaying the log_2_ intensities of individual proteins per group. Neutrophil elastase (NE), Myeloblastin (PRTN3), Cathepsin G (CTSG), Matrix metalloproteinases (MMP) 8 & 9. (**B**) Metalloproteinase inhibitor (TIMP) 1 & 2, α1-antichymotrypsin (SERPINA3), (**C**) Tree of the hematopoietic Stem Cell derived immune cell types, analyzed cell types are labeled in bold, average of the mean z-score of specific log_2_ protein intensity (left panel) and mean z-score of the different immune cell counts (right panel). Healthy controls (●); CF patients (●); COPD patients (●).

To provide a systems-wide view of disease-specific molecular alterations in CF and COPD and elucidate the underlying alterations contributing to these chronic respiratory diseases, we employed a mass spectrometry-based proteomics approach to a subset of our patient cohort (for cohort details see Supp. Table 2), for which a sufficient amount of material was left utilizing data-independent acquisition (DIA). A total of 1,495 proteins were identified and quantified across sputum supernatants from healthy individuals, CF patients, and COPD patients (Supp. Fig. 6B), with an average of 1,381 proteins detected per sample. Downstream data processing and bioinformatics analysis was performed using the MSPypeline to ensure robust and reliable statistical interpretation. Principal component analysis (PCA) of the proteomics data from healthy, CF, and COPD samples revealed significant differences between the proteomes of healthy individuals and CF patients. Strikingly, alterations in the sputum proteome of COPD patients were more heterogenous; for some patients, it overlapped with healthy profiles, for others with CF, and for most, it formed an intermediate group (Fig. 5A). These patterns highlighted that diverse molecular alterations may contribute to COPD. To characterize pathways dysregulated across these two muco-obstructive lung diseases, we first calculated global z-scores for the abundance of each protein across all samples (healthy, CF, and COPD) standardizing values relative to the cohort-wide mean. Proteins were then clustered based on their z-score patterns, yielding four distinct groups (Supp. Fig. 6 A). String Pathway Analysis was performed separately for each cluster revealing the following pathway associations: the adaptive immune system, O-linked glycosylation of mucins, protein targeting to membranes, and the matrisome (a collection of extracellular matrix proteins) To quantify pathway-level dysregulation, we calculated the mean z-scores for all proteins within each cluster. This approach summarized the collective deviation of pathway components from the global mean (Fig. 5B). The resulting proteomics profiles formed three sample clusters: one dominated by healthy controls, one dominated by COPD and one dominated by CF, mirroring the heterogeneity observed in the PCA. While in the cluster dominated by CF an upregulation of adaptive immune system and a downregulation of mucin glycosylation prevailed, in the cluster dominated by COPD exhibited a more heterogeneous response, characterized by a reduction in Signal Recognition Particle (SRP)-dependent protein targeting to the membrane and a trend towards a decrease in the matrisome (Fig. 5B). The comparison of the relative changes in proteins contributing to the top regulated pathways showed that for the three clusters, cluster 1 (dominated by healthy controls), cluster 2 (dominated by COPD patients) and cluster 3 (dominated by CF patients), characteristic changes in the balance of proteases and anti-proteases were observed. Specifically, for the pathway ‘protein targeting to membrane’ a trend towards changes in the proteases Trypsin-2 (PRSS2), Trypsin-3 (PRSS3) and Disintegrin and metalloproteinase domain-containing protein 9 (ADAM9) was identified, while for the ‘matrisome’ pathway changes in the balance of certain proteases and antiproteases prevailed (Fig. 6A,B). In particular for cluster 3 (CF dominated cluster), a strong upregulation of multiple proteases contributing to the pathway ‘adaptive immune system’ pathway (Fig. 6C) and a down regulation of many proteases and antiproteases associated with the pathway ‘O linked glycosylation of mucin’ pathway and of several mucins (Fig. 6D) was observed. To identify disease specific differences in the expression levels of key proteins, we compared the individual protein intensities. Our analysis showed that proteases such as neutrophil elastase (NE), proteinase 3 (PRTN3), and cathepsin G (CTSG), and matrix metalloproteinases (MMP) 8 and 9, were significantly more abundant in CF sputum and only increased to a lesser extent in COPD. Inhibitory proteins like α1-antichymotrypsin (SERPINA3) and TIMP1 were low in CF and intermediate in COPD, while TIMP2 was significantly elevated in CF while varying in COPD (Fig. 7A,B). However, it is important to note that CF sputum contains a highly proteolytic microenvironment^48^, evidenced by elevated neutrophil elastase activity (Fig. 7A), which may lead to proteolytic degradation of mucins and other proteins.

Thus, the identified disease specific changes in the sputum proteome indicate a key role for pathways leading to impaired mucus properties in CF and underscore the importance of tissue remodeling in COPD.

### Proteome-based cellular deconvolution points to a major contribution of eosinophils in COPD

Despite the common upregulation of neutrophils that we detected by cell counting in both chronic respiratory diseases, we observed major differences in the pattern of inflammatory factors present in the sputum of CF and COPD patients. To resolve this discrepancy, we tested whether we could utilize our detailed proteome-wide characterization of the sputum samples to deconvolute the cellular composition and focus in light of the major impact of inflammatory processes on the presence of immune cells. In analogy to the deconvolution algorithms and gold-standard datasets that have been developed for RNA sequencing data, we utilized curated marker lists from the Human Protein Atlas, ensuring that all selected markers are supported by proteomic evidence (Supp. Tab. 3 and Supp. Fig. 8). Applying this approach to our sputum proteomes, we estimated the relative contributions of immune cell populations. In line with the mean cell counts we determined (Fig. 7C right panel), the proteome-based deconvolution provided evidence for an upregulation of neutrophils and eosinophils in both chronic respiratory diseases and a downregulation of macrophages and B- and T-cells. Interestingly, these high-resolution examinations showed that while in CF the upregulation of neutrophils dominated, in COPD an increase in eosinophils prevailed (Fig. 7C left panel).

These findings demonstrate that, based on the global proteome information, the average cellular composition can be deduced and point to a distinct immune landscape in CF and COPD: While both chronic respiratory diseases are characterized by neutrophilia, COPD presents a more complex inflammatory profile with elevated eosinophils resulting in a unique cytokine signature, and revealing that fundamentally different alterations are indicative for these diseases.

### Integrative multi-omics factor analysis ranks contributions to disease phenotypes

**Fig. 8:**
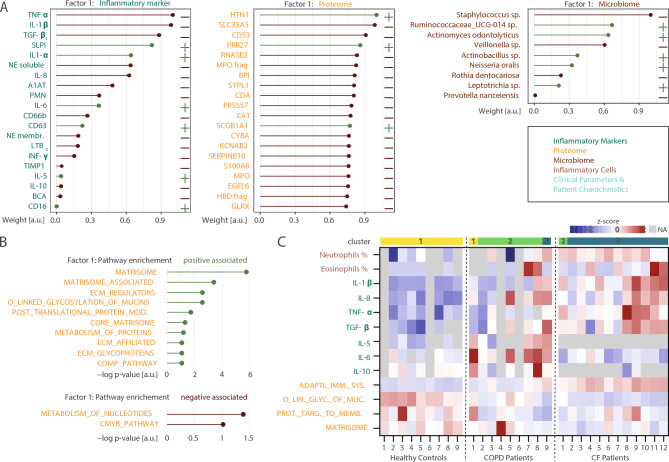
Multi-omics factor analysis of inflammatory markers, microbiome and proteomics in sputum from patients with CF, patients with COPD and Healthy controls. (**A**) Loadings (weights) of top features from the inflammatory marker (left), proteome (middle) and microbiome (right) of factor 1 from the MOFA. (**B**) Pathways enriched for the proteins (from the proteomic view) positively (left) or negative (right) correlated with factor 1. Enrichment p-values were calculated using gene set enrichment analysis (GSEA) against the human cancer hallmark gene sets from the molecular signatures database (MSigDB). (**C**) Heatmaps depicting the percentages of neutrophils and eosinophils, inflammatory markers (IL-1β, IL-8, TNF-α, TGF-β_1_, IL-5, IL-6, IL-10), and the top regulated pathways from the global proteome analysis (adaptive immune system, O-linked glycosylation of mucins, SRP-dependent cotranslational protein targeting to membrane, and the matrisome). The order of the patients per group is based on the PCA1 score from the global proteome analysis, the sample cluster of the individual samples is indicated on top. Heatmap cells with missing values are indicated depicted in light grey.

To gain insights into the complex interplay between microbial, molecular, and inflammatory processes in CF and COPD, an integration and comparison of the multi-level sputum data from healthy controls, CF patients, and COPD patients is required. Therefore, we applied MOFA, an unsupervised computational framework designed to integrate heterogeneous omics datasets^38^. MOFA enables the identification of key sources of variation across different molecular layers and ranks their relative impact, providing a systematic approach to uncovering disease-specific molecular signature and their contributions. Through integrating microbiome (13 species of 64 participants), proteomic (987 proteins of 30 participants), and inflammatory marker data (20 factors of 65 participants) (Supp. Fig. 9 A), MOFA identified six factors that explain at least 1% variance in any omic data (Supp. Fig. 9 B). Factor 1 was primarily driven by proteomic changes, but also reflected alterations in inflammatory markers and the microbiome. This factor showed a strong association with microbial classes and patient gender and was most effective in distinguishing healthy controls, CF patients, and COPD patients, making it the most predictive factor for the respective disease (Supp. Fig. 9D). Factor 2 was also informative for distinguishing CF and COPD as it was predominantly influenced by inflammatory markers and correlated with microbiome diversity metrics, including Shannon index, richness, evenness, and dominance (Supp. Fig. 9 C). The remaining factors played a less important role in differentiating CF from COPD (Supp. Fig. 9D). MOFA not only enables the identification of key diseases-specific patterns, but also allows for a detailed evaluation of the contribution of individual omic features and pathways regarding their direction and weight. Features with positive weights on a certain factor are those whose abundance are elevated in samples where this factor value is high while negative weights indicate a lower abundance in samples where the factor value is high. The absolute value of the weight of a individual feature indicates their importance. Factor 1 describes a gradient from healthy controls, COPD and CF with the factor values ranging from high to low. Deconstructing factor 1 provided evidence for distinct inflammatory and proteomic signatures in CF and COPD (Fig. 8A). Among inflammatory markers, TNF-α, IL1-β, and TGF-β_1_ were the most significant negative contributors, reflecting their higher abundance in CF patients compared to COPD patients and healthy controls. In contrast, SLPI, a key anti-inflammatory molecule, was the strongest positive contributor, indicating higher levels in COPD and healthy controls than CF patients. Within the proteome, histatin-1 (HTN1), proline-rich protein 27 (PRR27), and uteroglobin (SCGB1A1) were positive contributors, showing increased abundance in COPD and healthy controls. Conversely, negative contributors included solute carrier family 35 member A5 (SLC35A5), leukocyte surface antigen (CD53), and ribonuclease (RNASE2), which were more abundant in CF patients. In the microbiome, *Staphylococcus sp.* and *Veillonella sp.* emerged as the most negatively associated features, indicating its predominance in CF samples. Whereas *Ruminococcaceae_UCG-014 sp.* and *Actinomyces odontolyticus* had the strongest positive association, indicating a higher abundance in healthy control samples. Pathway enrichment analysis further highlighted the functional implications of factor 1. Positively associated proteins were enriched in the matrisome, extracellular matrix (ECM), and mucin-related pathways, underscoring their role in maintaining structural integrity and mucus properties which is impaired in patients compared to healthy controls. In contrast, negative associations were observed with nucleotide metabolism and the c-MYC pathway, suggesting distinct metabolic and cellular activity profiles in disease states (Fig. 8A). Interestingly, sorting of COPD patients based on their proteomic profiles with regards to similarities to healthy controls or CF patients revealed a gradient of increasing similarities to CF with respect to changes in cell counts, inflammatory factors and pathways, highlighting the heterogeneity of COPD patients and the possibility for subgrouping (Fig. 8B).

## Discussion

There is a growing clinical need for a reliable, and non-invasive method to assess lower airway health, particularly for millions of COPD patients, many of whom are in poor general health^49^. Traditional diagnostics, such as bronchoscopy or lung biopsies, are invasive, resource-intensive, and poorly tolerated, especially in severely ill or elderly patients. Therefore, developing accessible, patient-friendly diagnostic tools is crucial to improve disease monitoring, early intervention, and personalized treatment strategies. Building on the recognized value of sputum as a non-invasive sample, our SputOMICS workflow advances beyond conventional sputum analysis by employing high-throughput, data-independent acquisition mass spectrometry for deep, unbiased proteomic profiling of approximately 1,500 proteins from minimal samples. Integrating proteomics with microbial and inflammatory marker data enables comprehensive multi-omic profiling, and has the potential of enhancing disease stratification, pathway discovery, and biomarker identification. Semi-automated sample preparation reduces variability and supports scalability toward clinical implementation. To address this, we introduce the SputOMICs workflow, a robust multiomics pipeline that integrates clinical data, to uncover disease-specific microbiome, inflammatory, and proteomic signatures in the sputum. By systematically analysing molecular and cellular alterations, we revealed both shared and disease-specific molecular markers and pathways associated with CF and COPD pathophysiology, highlighting how sputum-based molecular profiling may support future efforts in patient stratification and inform the development of adjunctive therapeutic strategies. In this proof-of-concept study evaluating the potential of integrative multi-omics analyses of sputum samples, we deliberately chose to compare CF and COPD as two distinct muco-obstructive lung diseases of different etiologies to determine if this approach enables detection of differences that may not be visible when each disease is only compared to healthy controls. Notably, our data indicate the potential presence of COPD subgroups with distinct proteomic and inflammatory profiles that partially overlap with CF. Larger, independent studies are necessary to confirm improved classification and to explore opportunities for tailored interventions.

Sputum is an easily accessible clinical specimen, offering airway-specific insights by capturing inflammatory cells, microbes, and markers embedded in highly viscous mucus; providing greater diagnostic value than blood-based analyses and being easier to collect than BAL fluid. To ensure efficient sputum lysis and cellular integrity, we developed a standardized preparation protocol within the SputOMICs workflow, which separates sputum into two distinct fractions: the supernatant and the cell pellet, each offering complementary disease insights. The cell pellet retains viable neutrophils, enabling NE activity assessment and differential cell count via H&E staining, while the supernatant allows the analysis of microbiome composition, inflammatory markers, and the proteome, providing a comprehensive molecular and cellular profile of the airway environment. Notably, bulk proteomics-based cellular deconvolution aligned strongly with the H&E-based cell counts, despite being derived from different fractions (Fig. 7C). While sputum cytology remains the established standard for cell differential assessment, the proteomics-based deconvolution approach described here represents an emerging complementary method that enables simultaneous molecular and cellular profiling from the same sample. The clinical applicability and resolution of rare cell populations by proteomic deconvolution warrant further validation in larger studies. This agreement underscores the robustness of our workflow in accurately capturing the sputum cellular landscape and highlights proteomic deconvolution as a powerful alternative to traditional cell quantification methods.

Our microbiome analysis is in line with previously published studies and showed major difference between healthy and the two diseases groups but also uncovered striking differences between CF and COPD. The CF sputum was dominated by persistent pathogens such as *Pseudomonas aeruginosa* and *Staphylococcus*, which are already known to be strongly associated with neutrophilic inflammation, chronic infection and progressive lung damage. In contrast, COPD microbiomes were more diverse, often resembling healthy profiles or dominated by bacteria such as *Haemophilus*, *Streptococcus*, or *Lactobacillus. Haemophilus* was already described as a major driver of disease severity in COPD^22^*.*A key advantage of the integrated multi-omics approach is its potential to reveal mechanistic links between the airway microbiome and host proteomic responses, which enhances biological and clinical interpretation. For instance, the dominance of *Pseudomonas* in CF and *Haemophilus* in COPD strongly correlates with proteomic markers of inflammation and protease-antiprotease imbalance, suggesting that microbial dysbiosis actively drives distinct host immune and pathological states. This cross-omic synergy enables the identification of molecular subtypes within COPD characterized by unique microbiome-proteome profiles, reflecting disease heterogeneity that single-omic approaches cannot detect. Consequently, integrating these data layers deepens our understanding of disease mechanisms and supports more precise patient stratification, ultimately facilitating personalized therapeutic strategies.

In accordance with the chronic neutrophilic inflammation characteristic of CF and COPD, sputum from both disease harbours elevated neutrophil and eosinophil levels. However, proteome-based cellular deconvolution revealed a disease-specific shift: CF sputum presented a stronger neutrophilic increase, whereas COPD exhibited a predominance of eosinophils, potentially explaining their distinct inflammatory profiles. CF sputum displayed a more aggressive inflammatory phenotype, marked by pronounced neutrophilia, elevated IL-1β, TNF-α, TGF-β_1_, and reduced IFN-γ levels. In contrast, COPD sputum featured elevated IL-5, IL-6, and IL-10, suggesting a more heterogeneous inflammatory environment. These findings align with previous studies that identify CF as predominantly Th1-driven, while COPD inflammation reflects a mix of Th2 and regulatory responses^51,52^.

Both CF and COPD showed dysregulated protease-antiprotease activity, a hallmark of chronic respiratory diseases^2,3,36,43^. However, their distinct patterns highlight diverging pathophysiological trajectories. CF sputum showed markedly elevated NE activity and reduced SLPI levels, exacerbating proteolytic imbalance and airway damage. The highly proteolytic CF microenvironment likely leads to proteolytic degradation of mucins and other secreted proteins, which may influence their detectability and perceived abundance in proteomic analyses^53^. Other studies have demonstrated that while the overall abundance of matrix proteins in CF lung tissue may not differ significantly from controls, the diversity and specific composition of matrix proteins are altered, with the majority (> 90%) of individual matrix proteins expressed at lower levels in CF compared to control lung^54^ as a result from a chronic protease-antiprotease imbalance in CF airways where high level of neutrophil elastase and low levels of protease inhibitors are observed, leading to excessive breakdown of extracellular matrix components^55,56^. Conversely, while NE activity was also elevated in COPD, compensatory mechanisms, such as increased A1AT and NE/A1AT complexes, mitigates its effects.

The SputOMICs workflow allowed the robust identification of 1,500 proteins-a significant advancement in non-invasive respiratory disease diagnostics. Previous sputum proteomics studies have yielded valuable insights, yet with lower protein coverage; Yan et al*.* reported 280 proteins in COPD sputum, highlighting microbiome-host interactions^27^, while Maher et al*.* demonstrated CFTR modulator therapy-induced proteomic shifts, with 80 proteins increasing and 30 decreasing post-therapy^24^. Volpato et al*.* linked sputum rheology to eosinophilic inflammation, underscoring its potential as a disease biomarker^42^. Although tissue-based proteomics has provided critical insights—Ohlmeier et al*.* identified 82 altered proteins in lung tissue^19^, while Titz et al*.* and Schiller et al*.* characterized smoking-induced proteomic changes and lung injury repair, our study bridges the gap between tissue and airway proteomics. We identified CF-specific enrichment of adaptive immunity and mucin glycosylation proteins, while COPD sputum exhibited downregulation of SRP-dependent protein targeting and extracellular matrix components, reflecting impaired tissue remodelling. Those results are important in the context that it is often assumed that adaptive immunity is downregulated in CF due to the dominance of innate immune cells, particularly neutrophils, limiting the detection of adaptive immune signatures in sputum proteomic analyses^58^. Our results highlighting the specific enrichment of adaptive immunity is in line with observation showing a active engagement and specific alteration of T-cells and B-cells response rather than global suppression of innate immune responses^59–62^. Importantly, our deep proteomic coverage uncovered distinct COPD patient subgroups, with molecular profiles resembling either healthy controls or CF patients. These findings highlight the molecular heterogeneity within COPD detected through proteomic analysis, underscoring the potential of multi-omics approaches to support future disease classification and patient stratification efforts.

Integrating multi-omics data using MOFA provided key insights into the molecular and microbial drivers of disease phenotypes. Factor 1, primarily influenced by the sputum proteome, was strongly linked to microbial composition and inflammatory diversity, and most effectively distinguished healthy controls, CF, and COPD patients. We observed a gradient along this factor associated with neutrophilic inflammation, which is highly relevant for identifying subgroups within CF and COPD patients who might benefit from targeted anti-inflammatory interventions including emerging inhibitors of DPP-1/CatC to inhibit increased activity of all three neutrophil serine proteases (NE, PR-3 and CatG) in chronic neutrophilic inflammation^63,64^. Pathway enrichment analysis revealed CF-specific upregulation of mucin-related pathways. However, quantifying mucin proteins. by mass spectrometry remains challenging due to proteolytic degradation and detection limits, particularly in protease-rich environments in the CF airways. These technical limitations may affect the ability to accurately capture mucin abundance in proteomic datasets compared to other analytical methodologies. On the other hand, COPD showed alterations in matrisome composition and reductions in nucleotide metabolism as well as c-MYC pathways. These findings underscore the potential of integrated multi-omics approaches to uncover actionable biomarkers and improve patient stratification.

This study serves as a proof-of-concept for the potential of an integrative multi-omics approach, yet limitations exist. The relatively small sample size, single-center design, and age differences between CF and COPD cohorts may impact generalizability of our findings. We acknowledge that the use of spontaneous sputum for CF and COPD patients versus induced sputum for healthy controls represents a sampling method difference that may introduce biases related to sampling depth, mucus properties, and inflammatory cell profiles. However, prior studies have shown that while some cellular viability differences exist, sputum viscoelastic properties and key inflammatory markers remain broadly comparable between the two methods in chronic airway disease contexts^65–67^. Additionally, the cross-sectional design prevents causal inferences, emphasizing the need for longitudinal studies to validate findings and explore their clinical implications. Future research should focus on expanding cohorts, including longitudinal sampling, and validating the identified biomarkers to advance precision medicine for CF and COPD.

In summary, this study highlights the value of an integrative multi-omics approach in uncovering disease-specific microbiome, inflammatory and proteomic profiles in CF and COPD. Our findings provide a preliminary foundation for future research into disease subclassification and personalized therapeutic strategies, yet must be regarded as hypotheses generating until confirmed by large-scale validation studies linking molecular signatures to clinical endpoints. Future research, should prioritize validation of the sputum markers established by our workflow in larger, longitudinal cohorts and could enable the development of personalized interventions, enhanced subclassification of COPD as well as infection and inflammation in the CF patients that can be treated at the underlying protein defect with CFTR modulators^11,68,69^. Finally, our workflow could deepen the understanding of the relationship between microbiome, proteomic and inflammasome via easily acquired biomaterial like sputum enabling the analysis of larger patient cohorts. Validation of the finding in multi-center bigger cohorts could revolutionize the management and treatment of chronic respiratory disease and improve the outcomes for patients with CF and COPD.

## Supplementary Information

Supplementary Information.

## Data Availability

The datasets generated and/or analyzed during the current study are available in the proteomeXchange repository (https://proteomecentral.proteomexchange.org/ui; PXD048388) and the repository SRA (https://www.ncbi.nlm.nih.gov/sra; PRJNA1078153).
