# Supplementary material for: SputOMICs identifies common and distinct markers in cystic fibrosis and chronic obstructive pulmonary disease
Source: Sci Rep. 2025 Dec 24;15:44418. doi: 10.1038/s41598-025-32565-y (PMC12738762; doi:10.1038/s41598-025-32565-y)
Supplement: Supplementary file 1 — Supplementary Information. [file 41598_2025_32565_MOESM1_ESM.docx]

**SUPPLEMENT**

**Supplementary Materials and Methods**

**Study cohort**

Among the study participants, 41% of CF patients, 55% of COPD patients, and 50% of healthy controls were female. In terms of genotype, 14 CF patients were F508del homozygous, 20 were heterozygous, 4 had other CFTR mutations. Of the COPD patients, 12 were GOLD stage 3 (66%) and 6 were stage 4 (33%), they had a median residual volume (RV) of 208.5% predicted (ranging from 170.3-288.1% predicted), a median diffusing capacity of the lungs for carbon monoxide (Diff) of 34.15% predicted (ranging from 21.5-74.2% predicted), a median 6 minute walking distance of 328.5 m (ranging from 152-432 m), Tab. 1, Supp. Tab. 3 and Supp. Fig. 1A). The controls were volunteers with no history of respiratory disease.

**Sample preparation and workflow**

The supernatant fraction was used for measuring free activity, inflammatory parameters, proteomics, and microbiome analysis, while the cell pellet fraction was utilized for membrane NE activity and cell count assessments (Fig. 1).

**Free and surface associated neutrophil elastase activity**

Alterations in the balance between proteases and anti-proteases are key characteristics of chronic lung diseases progression ^1^. Free neutrophil elastase (NE) activity, a marker for CF progression, was measured using a previously established FRET-based assay to assess both soluble and membrane-bound NE ^2, 3, 4^ (Fig. 4A). Flow cytometry with neutrophil-specific gating was used to measure membrane-bound NE (surface markers are shown in Supp. Fig. 1C).

**Microbiota analysis**

Samples were freshly aliquoted (200μL) and treated with PMA^TM^ dye (Biotium Inc., Hayward, USA) to avoids subsequent PCR amplification of extracellular DNA from dead cells. 50 μM of PMA dye was added to the aliquot and incubated for 5 min in the dark. The samples were then exposed to light (650-Watt, 20 cm distance to the samples) on ice and shaking by 100 rpm for 5 min to cross-link PMA to DNA. Viable cells were pelleted by centrifugation at 5000 x g for 10 min. Supernatant was removed and cells were recovered in 200 μL of sterile PBS and stored at -20 °C until DNA extraction.

DNA extractions were performed using the QIAamp Mini Kit (QIAGEN, Hilden, Germany). Protease solution (7.2 mAU) and 200 μL of Buffer AL were added to the sample followed by a 15 sec vortex. Samples were incubated at 56 °C for 10 min and then purified according to the manufacturer’s protocol. DNA was eluted by adding 100 μL of buffer AE to the column, incubation for 1 min at room temperature and centrifugation at 6000 x g for 1 min. Negative controls were performed by doing the extraction without clinical samples.

DNA was amplified using universal bacterial primers flanking the V4 region (515F and 806R). Each primer was tagged with a spacer of 2-4 nucleotide and the sequencing adapter. Polymerase chain reactions (PCRs) were performed in 25 μL volumes composed of Q5 High-Fidelity 1X Master Mix (New England Biolabs GmbH, Germany), 25 pmol of each primer and 2 μL of DNA. Thermal cycling (Primus 25, Peqlab Biotechnologie GmbH, Germany or FlexCycler2, Analytik Jena AG, Germany) conditions were: first denaturation at 94 °C for 3 min, 30 amplification cycles (94 °C for 45 s, 50 °C for 1 min and 72 °C for 1 min 30 s) and a final extended cycle at 72 °C for 10 min. PCR products were purified using magnetic beads MagSi-NGSprep Plus (Steinbrenner Laborsystem GmBH, Wiesenbach, Germany) and processed through a second step amplification with primers targeting the sequencing adapter region and tagged with and individual barcodes and the illumine i5/i7 sequences. Each barcode is unique and differs by at least 3 nucleotides to all other barcodes to assign sequences to the samples. PCR products were purified with magnetic beads MagSi-NGSprep Plus (Steinbrenner Laborsystem GmBH, Wiesenbach, Germany) and quantified using the Quant-iT PicoGreen dsDNA Assay Kit (Thermo Fisher Scientific, Karlsruhe, Germany). Samples were pooled as an equimolar mix and sequenced on an Miseq system (Illumina Inc., San Diego, CA, USA) (600 cycles). PCR was also performed with negative controls from the extraction, and with sterile water to exclude contamination from the extraction kit and the PCR master mix. An internal control was performed by amplifying a mock community sample containing genomic DNA from 20 bacterial strains in equimolar (even) ribosomal RNA operon counts (HMD-782D, BEI Resources, ATCC, Manassas, VA, USA).

In parallel, the copy number of 16S rRNA gene was quantified by qPCR to evaluate the biomass using Unibac primer (forward: 5′-TGG AGC ATG TGG TTT AAT TCG A-3′; reverse: 5′-TGC GGG ACT TAA CCC AAC A-3′). PCR reactions were performed in 15 μL volumes composed of 1X Sybr-green mastermix (Life technology, Darmstadt, Germany), 50 pmol of each primer and 2 μL of DNA (or plasmid DNA standards). The thermal cycler conditions were: a first denaturation at 95 °C for 20 sec, 40 amplification cycles (95 °C for 3 sec, 60 °C for 30 sec) and two final steps at 95 °C for 15 sec and 60 °C for 1 minute followed by a melt curve. All reactions were performed in duplicate in a 7900HT Fast Real-time PCR system (Applied Biosystems, Foster City, USA). Quantification of the 16S number of copies was performed by comparison to the Cycle threshold value of a plasmid DNA standard which had been cloned in-house and quantified using Quant-iT PicoGreen dsDNA Assay Kit (Thermo Fisher Scientific, Karlsruhe, Germany).

The raw sequence data was processed using Dada2 to produce amplicon sequence variants (ASVs) with the following parameter: no ambiguities (N) allow, one error per reads allowed and truncation of the reads at the first position with a quality score < 2. Reads were merged as contigs and checked for chimera with the default parameters ^5^. The reads counts of each ASV was then used to create an ASV table and the sequenced were classified using Silva database v138.1. Those data were merged in a phyloseq object in R adding the metadata. No negative controls produce reliable amplicons and the Mock community positive control only produce amplicons assigned to the 20 expected species so we did not decontaminate our dataset. The mock community positive control was analysed to check for variability in the batch processing and we observed low variation between replicate with an average Morisita-horn distance of 0.008 (range: 0.003-0.014) and all the taxa expected to be present are detected but not as evenly as expected most likely due to amplification bias (Supp. Fig. 2). For each sample, a rarefaction curve was drawn to check that the coverage is correct (Supp. Fig. 3) and the bacterial community was characterized by calculating α-diversity (Shannon index), richness (number of ASV observed), evenness (Pielou index) and dominance (relative abundance of the most abundant ASV) and β-diversity (Morisita-Horn distance).

**Statistics**

Microbiome data were clustered using kmeans clustering based on the Morisita-Horn dissimilarity index. The optimal number of clusters was estimated based on the Gap statistic using the first SE max as threshold (Supp. Fig. 4). Spearman correlation was used to calculate the correlation between microbiota data, inflammatory markers and clinical data and was adjusted for multiple comparisons (Benjamini-Hochberg correction). Group wise comparisons were done with a pairwise Wilcoxon rank sum test adjusted for multiple comparisons. All statistical analyses were performed with R Statistical Software (v4.1.2; R Core Team 2021). A p-value <0.05 was considered statistically significant. Correlation between the microbiota and inflammatory markers or clinical data was done using Spearman correlation adjusted for multiple comparisons (Benjamini-Hochberg correction). The assessment of the microbiome by 16S RNA sequencing, via PCoA was based on the Unifrac weighted distance.

**Sample preparation for proteomic analysis**

Aliquots of stored samples were thawed at room temperature, centrifuged for 2 min at 12 000 x *g* at 4 °C, and the supernatant was transferred to a new vial. Total protein concentration was assessed by the BCA Assay (Pierce) and 5 µg of total protein was used per sample for further processing. Protein disulfide bonds were reduced with 40 mM Tris(2-carboxyethyl) phosphine hydrochloride (TCEP) and alkylated with 160 mM chloroacetamide at 95 °C temperature for 5 min. Protein digestion and clean-up were performed using an adapted version of the automated paramagnetic bead-based single-pot, solid-phase-enhanced sample-preparation (Auto-SP3) protocol ^6^ on the Bravo liquid handling platform (Agilent). Briefly, for bead preparation, Sera-Mag Speed Beads A and B (Ge Healthcare) were vortexed until the pellet was dissolved. The suspension was placed on a magnetic rack, and after one minute, the supernatant was removed. The beads were taken off the magnetic rack and suspended in water. This procedure was repeated three times. A total of 100 µL of bead A was combined with 100 µL of bead B, and the final volume was corrected to 100 µL with H_2_O. To each sample, a total of 5 µL of A+B beads were added. To induce the binding of the proteins to the beads, ethanol was added to each sample to a final 50% concentration (v/v). Samples were then incubated for 15 min at room temperature and 800 rpm. After the incubation step, samples were placed again on a magnetic rack, and after several minutes, the supernatant was removed. Samples were taken off the magnetic rack and washed in 80% ethanol. This procedure was repeated three times. Finally, samples were reconstituted in 100mM TEAB buffer containing trypsin (enzyme/protein ratio of 1:25) and digested overnight on a shaker at 37 °C and 1000 rpm. After digestion, the recovered peptides were dried down in a speed-vac and store at -80 °C until further analysis.

**LC-MS/MS measurements**

An Ultimate 3000 HPLC (Thermo Fisher Scientific, USA) was coupled to an Orbitrap Exploris 480 mass spectrometer (Thermo Fisher Scientific, Germany). For the full proteome analysis, peptides were dissolved in 15 µl loading buffer (0.1% formic acid (FA), 2% ACN in MS-compatible H_2_O), and 2 µL were injected for each analysis. The samples were loaded onto a pre-column (trap) at higher flowrates (PEPMAP 100 C18 5 µm 0.3 mm x 5 mm, Thermo Scientific), using a loading pump and then a valve was switched delivering the peptides to an analytical column (100 µm × 30 cm, packed in-house with Reprosil-Pur 120 C18-AQ, 1.9 µm resin, Dr. Maisch) at a flow rate of 3 µL/min in 98% buffer A (0.1% FA in MS-compatible H_2_O). After loading, peptides were separated using a 141 min gradient from 2% to 38% of buffer B (0.1% FA, 80% ACN in MS-compatible H_2_O) at 350 nL/min flow rate. The Orbitrap Exploris 480 was operated in data-independent (DIA) mode, with a m/z range of 350–1400. Full scan spectra were acquired in the Orbitrap at 120 000 resolution after accumulation to the set target value of 300% (100% = 1e6) and maximum injection time of 45ms. The full scans were followed by DIA scans. A total of 47 isolation windows were defined, with a m/z range of 406-986. DIA scan spectra were acquired at 30 000 resolution after accumulation to the set target value of 1000% (100% =1e5) and maximum injection time of 54ms. Normalized collision energy (NCE) was set to 28%.

**Database search and Data Analysis**

All DIA–MS data files were analyzed with a directDIA workflow using Spectronaut 15.6 (Biognosys, Zurich, Switzerland). For the Pulsar search, the UniProt Human-reviewed canonical reference proteome (downloaded on July 14^th^, 2020). The default settings for database match include: full specificity trypsin digestion, peptide length of between 7 and 52 amino acids and maximum missed cleavage of 2. N-terminal methionine was removed during preprocessing of the protein database. Carbamidomethylation at cysteine was used as a fixed modification, protein N-terminal acetylation and methionine oxidation were set as variable modifications. The false discovery rates (FDRs) were set as 0.01 for the peptide-spectrum match (PSM), peptide and protein identification. Other Spectronaut parameters for identification included maximum intensity as MZ extraction strategy, 0.01 precursor Qvalue Cutoff, 0.2 Precursor PEP Cutoff, stripped sequence as single hit definition. For quantification, identified (Qvalue) was set for precursor filtering, MaxLFQ as protein LFQ method and MS2 quantification with area as quantity type. For the cellular deconvolution, curated lists consisting of proteins which were found to be enriched in the corresponding cell types, i.e. Neutrophils (216), Eosinophils (72), Monocytes (126), T-Cells (336) and B-Cells (255) based on proteomic evidence were downloaded from the human protein atlas homepage (Supplementary Table 2) (<https://www.proteinatlas.org/>). Heatmaps comparing the three clusters were created using an updated version of the MSPypeline ^7^.

**Data Availability**

The original mass spectrometric raw data files along with the Spectronaut search result files are available on proteomeXchange ^8^, http://www.proteomexchange.org, under the accession number PXD048388.

**Multi-omics data integration**

All the data preprocessing and integrative analysis were executed in in R (version 4.2.0). Initially, each omic dataset underwent independent preprocessing steps. For the sputum proteomic data, The "PG.Quantity" column from the Spectronaut output file, containing protein quantity information, was imported into R. Subsequently, variance stabilizing normalization was applied using the 'vsn' package (version 3.64) ^9^. The microbiomic dataset was processed with the 'phyloseq' package (version 1.40) ^10^, incorporating the rarefy_even_depth function to address uneven sequencing depth among samples.

RNAseq counts underwent variance stabilizing transformation via the 'varianceStabilizingTransformation' function in the 'DESeq2' package (version 1.36.0) ^11, 12^. For the protein expression dataset of inflammatory markers, a straightforward log2 transformation was performed. To enhance the sensitivity of subsequent unsupervised multi-omic factor analysis, we prefiltered the sputum proteomic and microbiomic data, retaining only features significantly associated with disease status (raw P value < 0.05, ANOVA test).

### **Integrative multi-omics factor analysis**

The integration of the three pre-processed datasets (proteomic, microbiomic, and inflammatory marker expression) was conducted using the 'MOFA2' package (version 1.60). The integration process involved a variance threshold of 2% leading to the selection of 13 species from the microbiome of 64 participants, 987 proteins from 30 participants and 20 inflammatory markers from 65 participants. The "slow" convergence mode was used for the training of the model. A Gaussian noise model was employed for all three omic datasets. Subsequent exploration of integration results, including heatmap visualization and enrichment analysis, was carried out using utility functions within the 'MOFA2' package.

**Multiple comparison testing**

Gaussian distributed datasets were compared by Wilcoxon rank sum test with Bonferroni-Holm correction for multiple comparisons, whereas non-Gaussian distributed datasets were calculated with a Kruskal-Wallis ANOVA with a Dunnett's multiple comparison post-test. P-values are given as follows: *: p <0.05, **: p <0.01, ***: p <0.001 and ****: p <0.0001.

**Supplementary Figures**


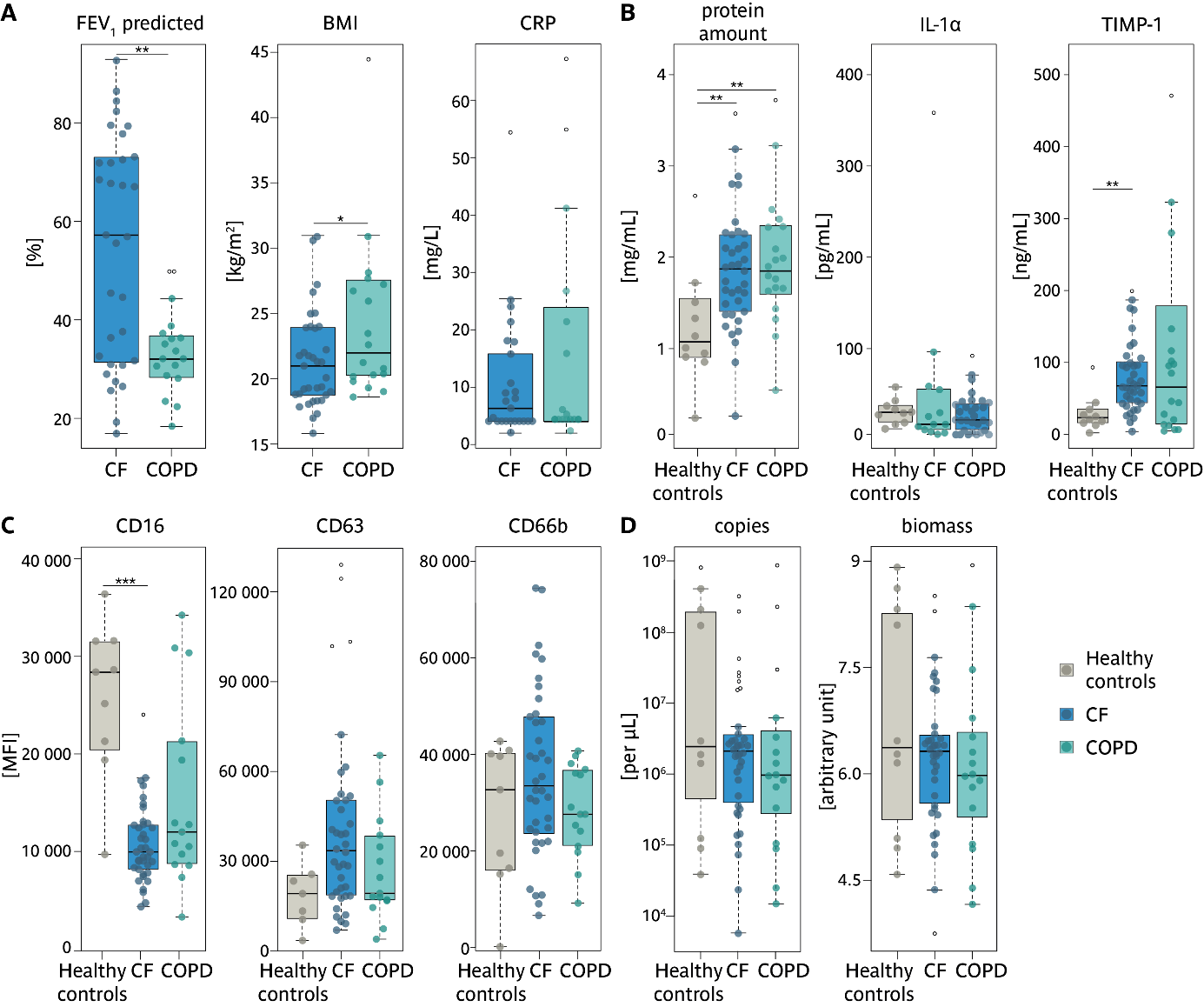


**Supplementary Figure 1: A** Comparison of clinical markers measured for COPD and CF patients only. Boxplots of lung function, forced expiratory volume in one second (FEV_1_) in percent predicted BMI and CRP. Healthy controls ● n=7; CF ● n=36; COPD ● n=14. **B**Comparison of the protein amount as well as additional inflammatory markers for healthy controls as well as COPD and CF patients. Boxplots of protein amount, IL-1α and TIMP1 given for each group. Healthy controls ● n=10/10/10; CF ● n=38/31/37; COPD ● n=18/13/18. **C**Comparison of surface-markers CD16, CF63 and CD66b of the neutrophils isolated from sputum of healthy controls as well as COPD and CF patients. Boxplots of CD16 and CD63 and CD66b mean fluorescent intensity (MFI). Healthy controls ● n=9/7/9; CF ● n=38/38/38; COPD ● n=15/15/15. **D** Additional microbiome parameters for the healthy controls as well as COPD and CF patients. Boxplots of copies per µL biomass of each group. Healthy controls ● n=10; CF ● n=38; COPD ● n=16. Outliers are shown as empty circles (**○**).


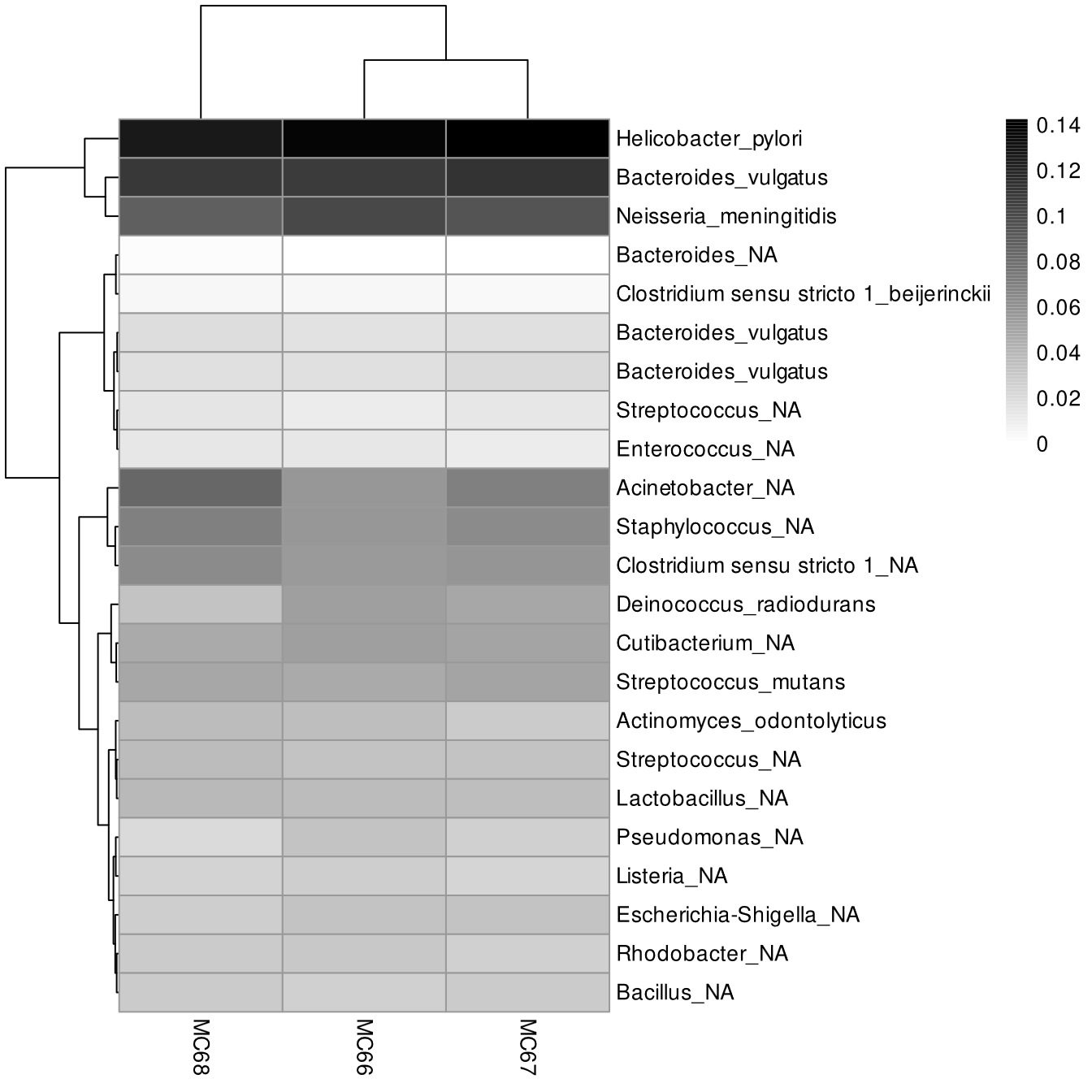


**Supplementary Figure 2: Microbiome composition of the mock community.**


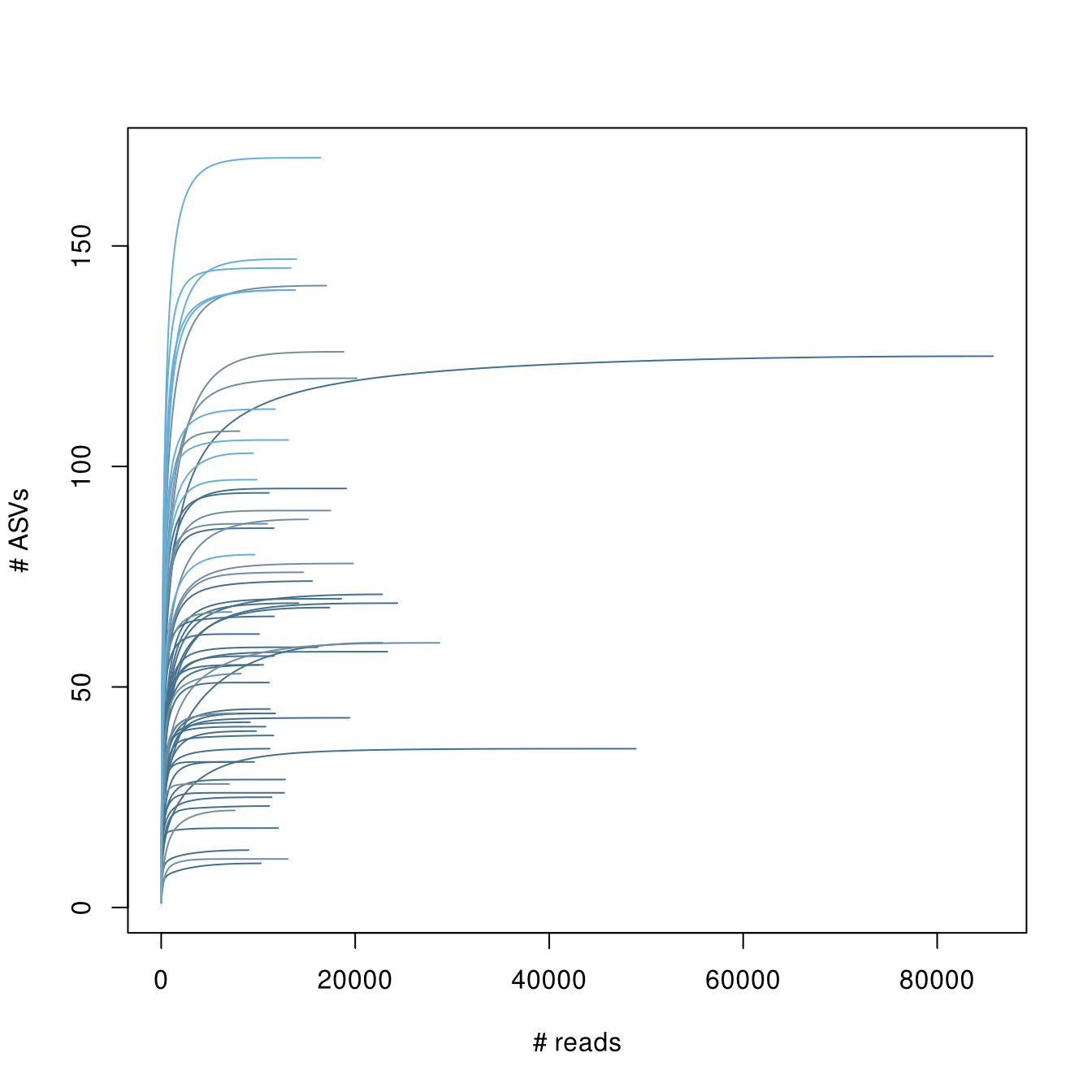


**Supplementary Figure 3: Rarefaction curve,** number of reads vs number of ASVs.


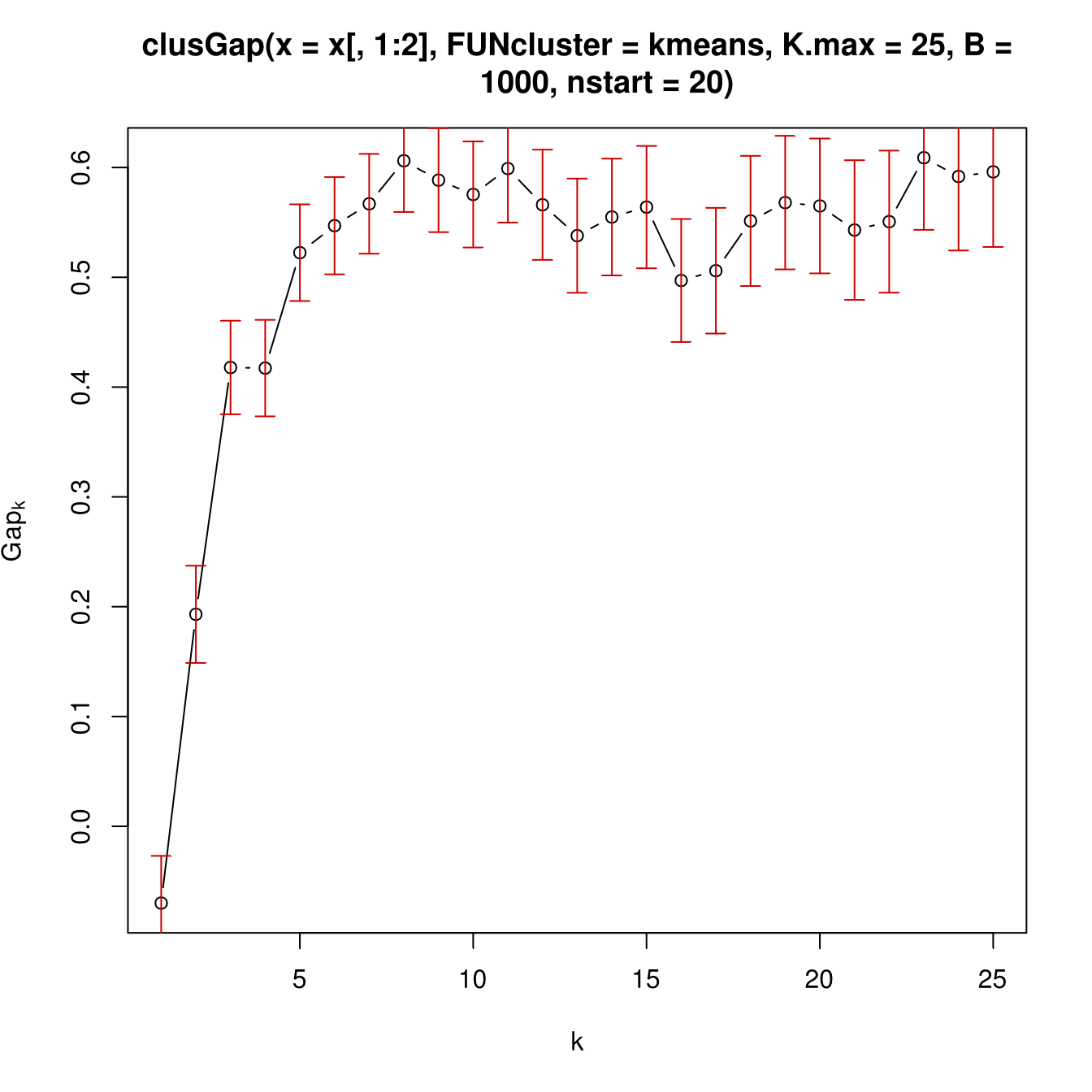


**Supplementary Figure 4: Gap statistic.** Based on the function clusGap(x=x[, 1:2], FUNcluster=kmeans, K.max=25, B=1000, nstart=20, according to Tibshirani et al. ^13^.


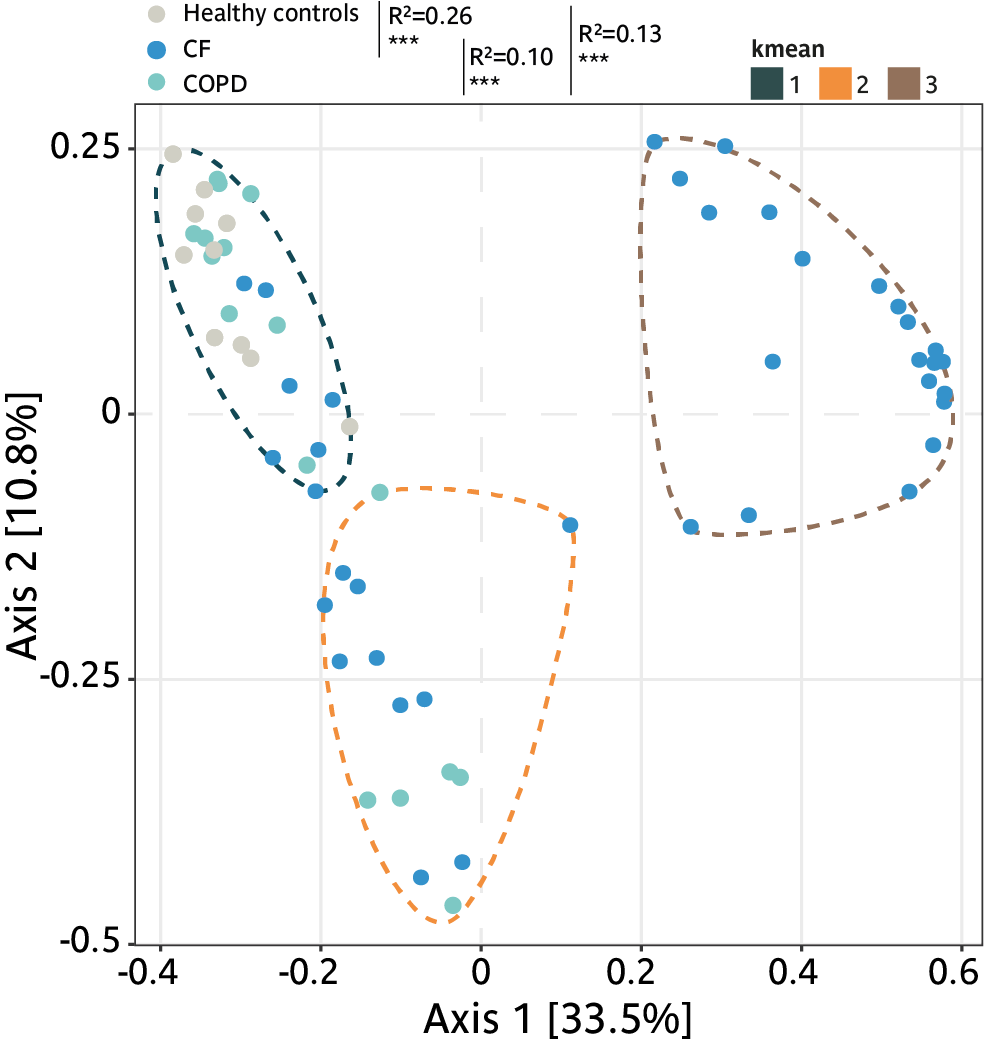


**Supplementary Figure 5: Principal coordinate analysis (PCoA) of the microbiome** based on a Unifrac weighted distance matrix, healthy controls; CF; COPD are indicated by color, healthy controls (●); CF patients (●) and COPD patients (●). The kmean cluster are indicated with the dashed lines, in teal, orange and brown.

**
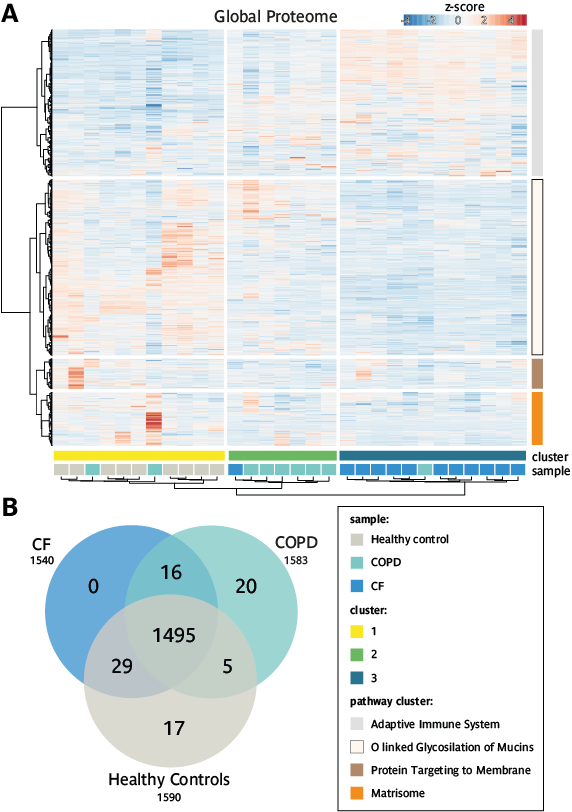
**

**Supplementary Figure 6: Detailed analysis of the proteomic measurements. A** Heatmap of the Global proteome, with the four protein clusters based on the Ward’s method indicated. **B**Venn diagram of all proteins identified in the three groups; Healthy controls, CF and COPD patients.


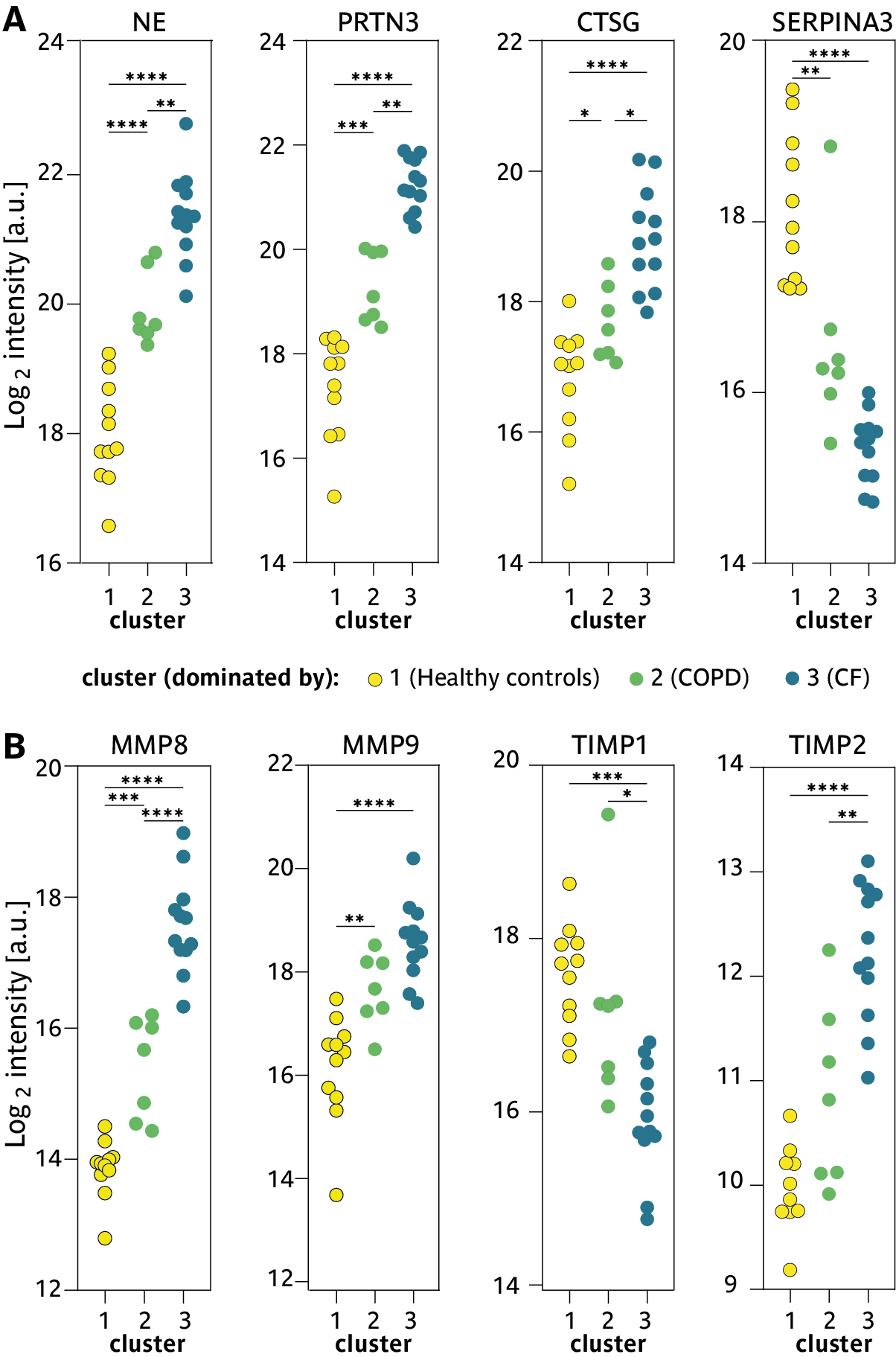


**Supplementary Figure 7: Detailed analysis of specific proteins of the proteomic measurements.** Confetti plots displaying the log_2_ intensities of individual proteins per sample based on the cluster dominated by one disease group. **A** Neutrophil elastase (NE), Myeloblastin (PRTN3), Cathepsin G (CTSG), α1-antichymotrypsin (SERPINA3), **B** Matrix metalloproteinases (MMP) 8 & 9, Metalloproteinase inhibitor (TIMP) 1 & 2, dominated by Healthy Controls (●); cluster 2, dominated by COPD patients (●) and cluster 3, dominated by CF patients (●).


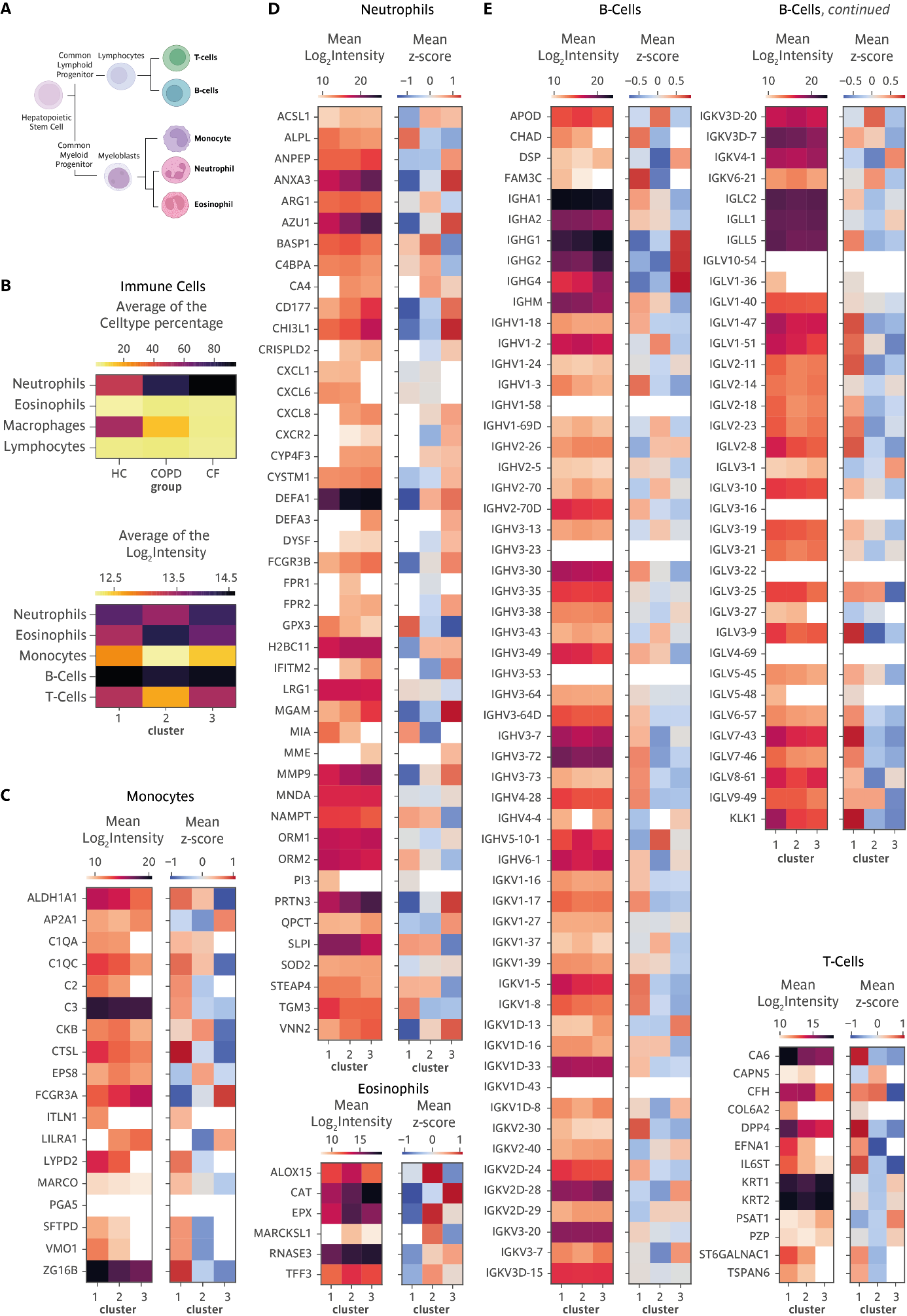


**Supplementary Figure 8: Immune Cell types per sample cluster of the global sputum proteomics measurements. A** Tree of the hematopoietic Stem Cell derived immune cell types, analyzed cell types are labeled in bold **B** Average of mean z-score of the different immune cell types by cell counting and based on the abundance of signature proteins. **C** Mean Log_2_ intensity and mean z-score of Monocyte specific proteins. **D** Granulocyte specific mean Log_2_ intensity and mean z-score of Neutrophil and Eosinophil specific proteins. **E** Lymphocyte specific mean Log_2_ intensity and mean z-score of B-cell and T-cell specific proteins.


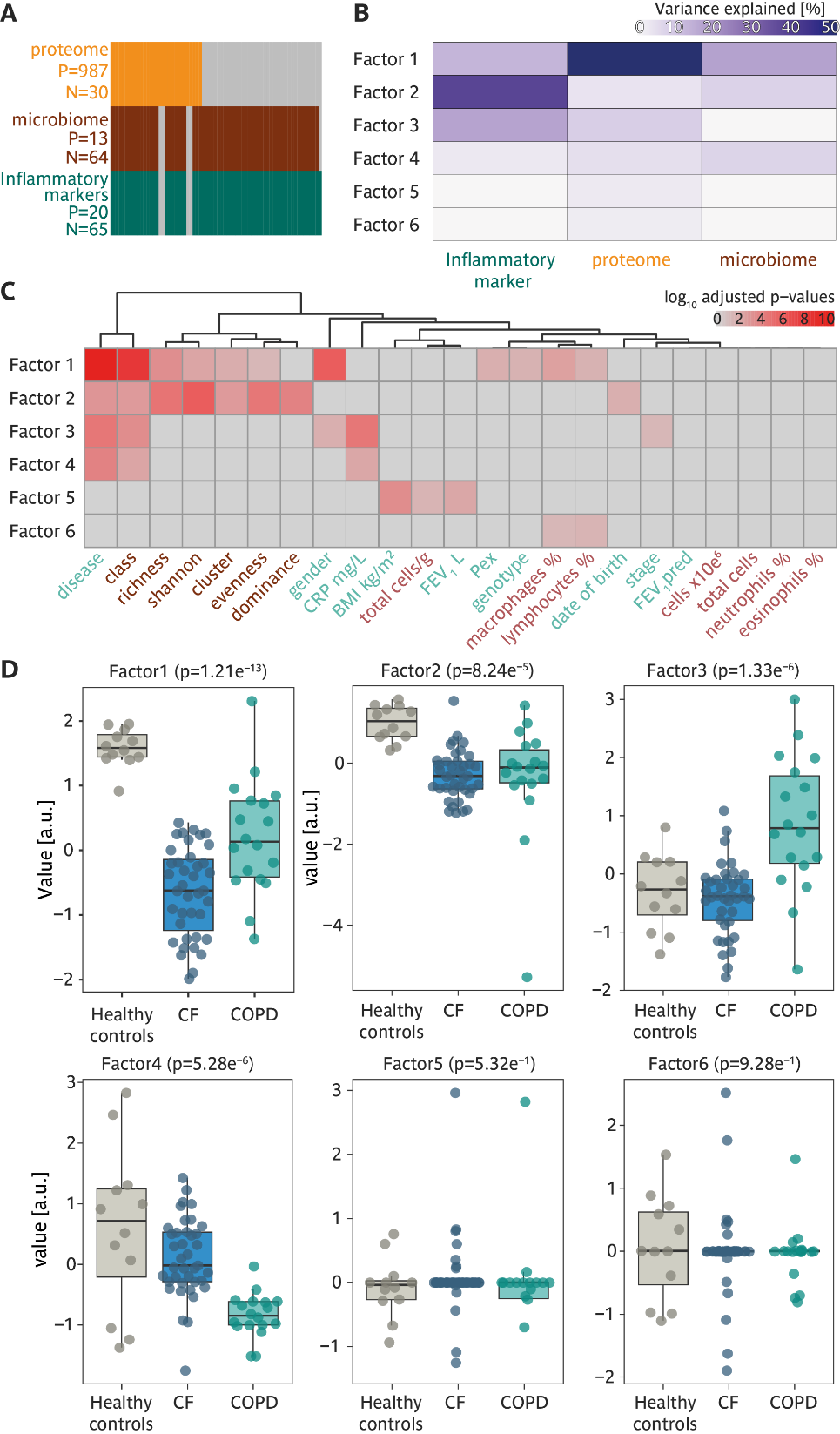


**Supplementary Figure 9: Multi-Omics Factor Analysis of ELISA, Microbiome and Proteomics Measurements of the healthy controls as well as CF and COPD patients. A**Overview of the input datasets for MOFA. Grey blocks indicate the dataset (view) for certain samples are not available. **B** Explained variance for each omic dataset by each MOFA factor. **C** Heatmap showing the association p-values between MOFA factors and sample metadata. For continuous metadata features (e.g. BMI, CRP, total cells etc.), p-values were calculated using Pearson’s correlation tests. For categorical metadata features (e.g. gender, stage and so on), ANOVA test was used. B Boxplot showing the distribution of factor 1 values in the Healthy controls, CF patients, and COPD patients. P-value was calculated by ANOVA test.  **D** Boxplots showing the distribution of Factor 1 to 6 among the three groups, healthy controls (●); CF patients (●) and COPD patients (●).

**Supplementary Tables**

**Supplementary Table 1: Number of included measurements of the inflammatory markers.** Smallest group sizes are indicated in bold.

|  | **Healthy controls** | **CF** | **COPD** |
| --- | --- | --- | --- |
| **IL-1β** | 10 | 38 | 18 |
| **TNF-α** | 10 | 38 | 17 |
| **TGF-β_1_** | 10 | 35 | 17 |
| **IL-8** | 10 | 38 | 18 |
| **LTB_4_** | 10 | **31** | 17 |
| **IL-5** | **8** | 32 | **13** |
| **IL-6** | 10 | 38 | 10 |
| **IL-10** | 10 | 36 | 11 |
| **INF-γ** | 10 | 33 | 11 |

**Supplementary Table 2: Demographics and clinical characteristics of study subpopulation analyzed by proteomics**

|  |  | **CF** | **COPD** | **Healthy controls** |
| --- | --- | --- | --- | --- |
| **Subjects** | n | 12 | 10 | 9 |
| **Age (years)** | Median  (range) | 27.36  (21.88-37.61) | 65.03  (51.78-78.20) | 31.45  (27.94-49.04) |
| **Sex** | n, females/ males | 4/8 | 4/6 | 4/5 |
| **BMI (kg/m²)** | Median  (range) | 19.25  (17.27-30-96) | 20.78  (18.59-44.46) | - |
| **FEV_1_ % predicted*** | Median  (range) | 70.30  (26.58-86.61) | 39.27  (32.88-57.90) | - |
| **CFTR genotype** |  |  |  |  |
| F508del/F508del | n (%) | 7 (58.33) | - | - |
| F508del/other | n (%) | 4 (33.33) | - | - |
| Other/other | n (%) | 1 (8.33) | - | - |
| **Pancreatic insufficiency** | n (%) | 6 (50.00) | - | - |
| **GOLD stage (3/4)** | n (%) | - | 7(70.00)/3 (30.00) | - |
| **RV % predicted** | Median  (range) | - | 224.60  (170.3 – 288.1) | - |
| **Diff % predicted** | Median  (range) | - | 37.55  (24.00 – 74.20) | - |
| **6 minute walking test (m)** | Median  (range) | - | 322.50  (152.00 – 432.0) | - |

*FEV_1_ % predicted only available from 10 CF patients of the subset

**Supplementary Table 3: List of signature proteins used for cellular deconvolution, retrieved form the Human Protein Atlas.**

| **Monocytes** | | **Neutrophils** | | | | | | | **Eosinophils** | | | **B-Cells** | | | | | | | | | **T-Cells** | | | | | | | | | | |  |  |
| --- | --- | --- | --- | --- | --- | --- | --- | --- | --- | --- | --- | --- | --- | --- | --- | --- | --- | --- | --- | --- | --- | --- | --- | --- | --- | --- | --- | --- | --- | --- | --- | --- | --- |
| ABCC3 | NEURL1 | | | AATK | | FAM53C | | PHF24 | | | ADORA3 | | | ABCB4 | | IGHV1-58 | | IGLC2 | | SLC26A4 | | | ABCD2 | | EPHX2 | | MID2 | | SLC44A3 | | TRBV6-7 | | |
| ABCC6 | NR4A1 | | | ABTB1 | | FCGR2A | | PI3 | | | ALOX15 | | | AICDA | | IGHV1-69-2 | | IGLC3 | | SLC2A5 | | | ACER1 | | EPHX4 | | MLF1 | | SLC4A10 | | TRBV7-1 | | |
| ACP2 | NRG1 | | | ACSL1 | | FCGR3B | | PIP4P2 | | | AOC1 | | | AIM2 | | IGHV1-69D | | IGLL1 | | SLC38A11 | | | ACSL6 | | FAAH2 | | MMP28 | | SNPH | | TRBV7-3 | | |
| ALDH1A1 | OLFML2B | | | ADARB2 | | FOS | | PITPNM3 | | | ARHGEF33 | | | AKAP6 | | IGHV2-26 | | IGLL5 | | SNX22 | | | ACTG2 | | FAIM2 | | MORC2 | | SOAT2 | | TRBV7-9 | | |
| AP2A1 | PALM | | | ADM | | FPR1 | | PKN2 | | | ASB2 | | | APBB2 | | IGHV2-5 | | IGLV10-54 | | SOBP | | | ADAM12 | | FAM149A | | MPRIP | | SPOCK2 | | TRBV9 | | |
| APOBEC3A | PAQR4 | | | ALDH1A2 | | FPR2 | | PLIN5 | | | AVP | | | APOD | | IGHV2-70 | | IGLV1-36 | | SOX5 | | | ADAM23 | | FAM167B | | MT3 | | SPON1 | | TRDV1 | | |
| ARHGAP29 | PCDH12 | | | ALPL | | G0S2 | | PLPPR2 | | | CAMK1 | | | ARL14 | | IGHV2-70D | | IGLV1-40 | | SPACA3 | | | ADGRG1 | | FAM169A | | MTUS1 | | SPON2 | | TRDV2 | | |
| AVPR2 | PGA4 | | | ANGPT1 | | GALNT14 | | POLGARF | | | CAT | | | ARL4D | | IGHV3-11 | | IGLV1-44 | | SSPN | | | ADIRF | | FAM171A1 | | MXRA8 | | SSTR3 | | TRDV3 | | |
| C1orf53 | PGA5 | | | ANKRD34B | | GK | | PPP1R12B | | | CCL23 | | | BANK1 | | IGHV3-13 | | IGLV1-47 | | STEAP1B | | | ADTRP | | FAM241B | | NEDD4L | | ST6GALNAC1 | | TRGJ1 | | |
| C1QA | PLAGL2 | | | ANPEP | | GLT1D1 | | PROK2 | | | CCL4L2 | | | BEND5 | | IGHV3-20 | | IGLV1-50 | | STK33 | | | APBA2 | | FANK1 | | NEFL | | STMN3 | | TRGV10 | | |
| C1QC | POFUT1 | | | ANXA3 | | GNG10 | | PRRG4 | | | CEBPE | | | BHLHE41 | | IGHV3-23 | | IGLV1-51 | | SYBU | | | APBB1 | | FBLN7 | | NELL2 | | SUSD4 | | TRGV11 | | |
| C2 | PPM1N | | | AOC2 | | GPX3 | | PRTN3 | | | CFAP276 | | | BLK | | IGHV3-30 | | IGLV2-11 | | SYNPO | | | AQP3 | | FBXO15 | | NMU | | SYTL2 | | TRGV2 | | |
| C3 | PPP1R17 | | | AP5B1 | | H1-6 | | PTGS2 | | | CHST13 | | | CCN2 | | IGHV3-33 | | IGLV2-14 | | SYT1 | | | B3GALT2 | | FEZ1 | | NOG | | TAFA1 | | TRGV3 | | |
| CABP4 | RHOC | | | AQP9 | | H2AC19 | | PVALB | | | CYSLTR2 | | | CD19 | | IGHV3-35 | | IGLV2-18 | | TAL2 | | | B3GAT1 | | FGFBP2 | | NOS3 | | TARP | | TRGV4 | | |
| CCDC149 | RIN1 | | | ARG1 | | H2BC11 | | QPCT | | | DIXDC1 | | | CD200 | | IGHV3-38 | | IGLV2-23 | | TAS1R3 | | | BAG3 | | FHIT | | NPDC1 | | TCEA3 | | TRGV8 | | |
| CCL8 | RRAS | | | ARHGAP28 | | H2BC18 | | R3HDM4 | | | ECHDC3 | | | CD22 | | IGHV3-43 | | IGLV2-33 | | TEAD2 | | | BATF | | FLT3LG | | NR3C2 | | TEPP | | TRGV9 | | |
| CD300E | RXFP2 | | | ASF1B | | H2BC4 | | RAB36 | | | EPX | | | CD72 | | IGHV3-48 | | IGLV2-8 | | TEX9 | | | BCL11B | | FLT4 | | NRCAM | | THEM5 | | TRIM16 | | |
| CD300H | S1PR3 | | | ASPRV1 | | H3-7 | | RALBP1 | | | F8A2 | | | CD79A | | IGHV3-49 | | IGLV3-1 | | TLR10 | | | BEX3 | | FOXB1 | | NSG1 | | THEMIS | | TRIM69 | | |
| CDH23 | SASH1 | | | ATG2A | | H4C2 | | RASSF3 | | | F8A3 | | | CD79B | | IGHV3-53 | | IGLV3-10 | | TNFRSF13B | | | BFSP2 | | FOXP3 | | NTN4 | | TIGIT | | TROAP | | |
| CDKN1C | SCUBE2 | | | ATP13A5 | | HCAR2 | | RFX2 | | | FAM170B | | | CD81 | | IGHV3-64 | | IGLV3-16 | | TNFRSF13C | | | BIRC5 | | GFPT2 | | OGN | | TIMD4 | | TSHZ2 | | |
| CDKN2B | SEMA3A | | | ATP23 | | HDAC7 | | RNF149 | | | FHL3 | | | CELSR1 | | IGHV3-64D | | IGLV3-19 | | TUBB3 | | | BNC2 | | GJB6 | | OXNAD1 | | TJP3 | | TSPAN15 | | |
| CDYL2 | SFTPD | | | AVIL | | HEY1 | | RPS4Y2 | | | FOXI1 | | | CEP295NL | | IGHV3-66 | | IGLV3-21 | | UGT2B17 | | | BOLL | | GLB1L3 | | PAGE5 | | TM4SF19 | | TSPAN6 | | |
| CKB | SIDT2 | | | AZU1 | | HGD | | RSPH14 | | | FRRS1 | | | CHAD | | IGHV3-7 | | IGLV3-22 | | UGT8 | | | C20orf204 | | GPA33 | | PAK3 | | TMEM116 | | TTC16 | | |
| CLEC5A | SLC18A1 | | | B3GNT5 | | HSPA6 | | SBNO2 | | | GADD45G | | | CHL1 | | IGHV3-72 | | IGLV3-25 | | USP6NL | | | CA6 | | GPR171 | | PARVA | | TMEM132C | | TTC39C | | |
| CNTLN | SLC24A4 | | | B3GNTL1 | | HYCC2 | | SDC2 | | | GAPT | | | CLEC17A | | IGHV3-73 | | IGLV3-27 | | VPREB1 | | | CACNB3 | | GRAP2 | | PBX4 | | TMEM171 | | UBASH3A | | |
| CRYBB1 | SLC45A1 | | | BASP1 | | ID1 | | SEC14L1 | | | GATA1 | | | CNFN | | IGHV4-28 | | IGLV3-9 | | VPREB3 | | | CAMK4 | | GSC | | PCLAF | | TMEM204 | | ULBP2 | | |
| CSF1R | SLC46A2 | | | BEST1 | | IFITM2 | | SHOC1 | | | GSTM4 | | | CNTNAP2 | | IGHV4-31 | | IGLV4-3 | | WNT16 | | | CAPN5 | | GTSF1L | | PCSK1N | | TMEM255A | | UNC45B | | |
| CTSK | SLC5A10 | | | BMX | | IL1B | | SLC19A1 | | | HAS1 | | | COCH | | IGHV4-4 | | IGLV4-60 | | ZBTB32 | | | CCDC57 | | GZMH | | PDCD1 | | TMIE | | UTS2 | | |
| CTSL | SLC7A7 | | | BTNL3 | | IL1R2 | | SLC22A1 | | | HES1 | | | COL19A1 | | IGHV4-59 | | IGLV4-69 | | ZNF318 | | | CCDC65 | | HACD1 | | PDGFB | | TMPRSS3 | | VIT | | |
| CTTNBP2 | SOX15 | | | BTNL8 | | INKA2 | | SLC22A4 | | | HES4 | | | COL4A3 | | IGHV4-61 | | IGLV5-37 | | ZNF860 | | | CCL20 | | HAPLN3 | | PI16 | | TNFRSF25 | | VSIG1 | | |
| CXCL10 | SPIC | | | C17orf107 | | IRAG1 | | SLC25A37 | | | HES6 | | | COL4A4 | | IGHV5-10-1 | | IGLV5-45 | |  | | | CCNB2 | | HKDC1 | | PIK3IP1 | | TNFRSF4 | | WNT1 | | |
| CXCL2 | SPRED1 | | | C3orf62 | | KAZN | | SLC26A8 | | | IDO1 | | | CORO2B | | IGHV6-1 | | IGLV5-48 | |  | | | CCR4 | | HOXC5 | | PKIA | | TNIP3 | | WNT7A | | |
| CYP1B1 | TAGLN | | | C4BPA | | KCNJ15 | | SLC6A6 | | | IL34 | | | CPNE5 | | IGHV7-4-1 | | IGLV5-52 | |  | | | CCR7 | | HPCAL4 | | PKMYT1 | | TNNC1 | | ZCCHC12 | | |
| CYSRT1 | TBC1D8 | | | CA4 | | KCNJ16 | | SLPI | | | KCNJ8 | | | CR2 | | IGHV7-81 | | IGLV6-57 | |  | | | CCR8 | | HSD11B1 | | PLCL1 | | TPBG | | ZNF683 | | |
| DENND2B | TCF7L2 | | | CACNB4 | | KCNJ2 | | SMCHD1 | | | KHDRBS3 | | | CXCR5 | | IGKV1-16 | | IGLV7-43 | |  | | | CD248 | | HYKK | | PLEK2 | | TPO | | ZNF80 | | |
| EDN1 | TCN2 | | | CALD1 | | KIAA0825 | | SOD2 | | | LGALS12 | | | DBNDD1 | | IGKV1-17 | | IGLV7-46 | |  | | | CD28 | | ICA1 | | PLEKHB1 | | TRABD2A | | ZWINT | | |
| EGR2 | TDRD9 | | | CBS | | KIF27 | | SOX6 | | | LIMS3 | | | DKKL1 | | IGKV1-27 | | IGLV8-61 | |  | | | CD3D | | ICOS | | PLK1 | | TRAC | |  | | |
| ELAVL4 | TESC | | | CCDC153 | | LETM2 | | SPP1 | | | LRRC17 | | | DNAJC5B | | IGKV1-33 | | IGLV9-49 | |  | | | CD3G | | IFNG | | PLLP | | TRAF1 | |  | | |
| EPB41L3 | THAP10 | | | CD177 | | LITAF | | SRXN1 | | | LYPD1 | | | DSP | | IGKV1-37 | | IL5 | |  | | | CD40LG | | IKZF4 | | PLPP2 | | TRAV10 | |  | | |
| EPS8 | TMEM176A | | | CD300LD | | LRG1 | | ST6GALNAC2 | | | MARCKSL1 | | | DYNC2I2 | | IGKV1-39 | | IL6 | |  | | | CD5 | | IL23R | | PM20D1 | | TRAV12-1 | |  | | |
| ERRFI1 | TMEM176B | | | CDH17 | | LRRC4 | | STEAP4 | | | MARK3 | | | E2F5 | | IGKV1-5 | | ILDR1 | |  | | | CD6 | | IL32 | | PMCH | | TRAV12-2 | |  | | |
| FBN2 | TNFRSF8 | | | CEACAM3 | | LRRK2 | | SVIL | | | MGAT4B | | | EBF1 | | IGKV1-6 | | JSRP1 | |  | | | CD8A | | IL6ST | | PODN | | TRAV12-3 | |  | | |
| FCGR3A | TNS1 | | | CFAP58 | | LY6G6F | | TECPR2 | | | MYCT1 | | | EHD3 | | IGKV1-8 | | KCNG1 | |  | | | CD8B | | ISM1 | | PPP1R3F | | TRAV13-1 | |  | | |
| FIBCD1 | TTYH3 | | | CFAP92 | | LY6K | | TEKT2 | | | NAA80 | | | ELL3 | | IGKV1-9 | | KCNH8 | |  | | | CDO1 | | ITGAE | | PRG4 | | TRAV14DV4 | |  | | |
| FMNL2 | ULK2 | | | CFLAR | | LY96 | | TEX54 | | | OLIG2 | | | ENSG00000275063 | | IGKV1D-12 | | KLHL14 | |  | | | CEP55 | | ITPKA | | PRSS35 | | TRAV17 | |  | | |
| FXYD6 | UTRN | | | CHI3L1 | | MADCAM1 | | TGM3 | | | PDE4D | | | FAM177B | | IGKV1D-13 | | KLK1 | |  | | | CFAP95 | | ITPRIPL1 | | PSAT1 | | TRAV19 | |  | | |
| FZD1 | VMO1 | | | CMTM2 | | MAK | | TLE3 | | | PIP5K1B | | | FAM3C | | IGKV1D-16 | | LARGE2 | |  | | | CFH | | JAKMIP1 | | PTCH1 | | TRAV20 | |  | | |
| GPBAR1 | WDR62 | | | CNTNAP3 | | MANSC1 | | TLR6 | | | PNMA6A | | | FBXO10 | | IGKV1D-17 | | LCN10 | |  | | | CHN1 | | KALRN | | PTTG1 | | TRAV21 | |  | | |
| GPNMB | XYLT1 | | | CNTNAP3B | | MAP1LC3A | | TMCC1 | | | PPDPF | | | FCER2 | | IGKV1D-33 | | LIX1 | |  | | | CHODL | | KCNA2 | | PZP | | TRAV22 | |  | | |
| GPR20 | ZG16B | | | CREB5 | | MBD6 | | TMEM252 | | | PPP1R1B | | | FCRL1 | | IGKV1D-37 | | LTBP3 | |  | | | CIART | | KIAA1671 | | RAB25 | | TRAV24 | |  | | |
| GSTA4 | ZNF703 | | | CRISPLD2 | | MGAM | | TNFAIP6 | | | PPP2R5B | | | FCRL2 | | IGKV1D-39 | | MAJIN | |  | | | CLIC5 | | KIF19 | | RAB6B | | TRAV26-1 | |  | | |
| GUCY1B1 |  | | | CSAD | | MIA | | TNFRSF10C | | | PRKCZ | | | FCRL5 | | IGKV1D-42 | | MARCHF3 | |  | | | COL6A2 | | KIR3DL1 | | RAI2 | | TRAV27 | |  | | |
| HMOX1 |  | | CSF3R | | MIDN | | TP53INP2 | | | PRSS33 | | | FCRLA | | IGKV1D-43 | | MS4A1 | |  | | | COLQ | | KLRG1 | | RASGRF2 | | TRAV29DV5 | |  | | |  |
| ICAM4 |  | | CSNK1A1L | | MKLN1 | | TRANK1 | | | PYROXD2 | | | FNDC11 | | IGKV1D-8 | | MYL2 | |  | | | CPA5 | | KRT1 | | RASGRP1 | | TRAV30 | |  | | |  |
| IL10 |  | | CXCL1 | | MME | | TREM1 | | | RNASE3 | | | FRMD4A | | IGKV2-24 | | NIPAL4 | |  | | | CPNE7 | | KRT2 | | RBM11 | | TRAV4 | |  | | |  |
| INSIG1 |  | | CXCL6 | | MMP9 | | UBN1 | | | RPS6KA2 | | | GLYATL1B | | IGKV2-28 | | NTNG1 | |  | | | CTLA4 | | KRT73 | | RCAN2 | | TRBC1 | |  | | |  |
| ITLN1 |  | | CXCL8 | | MNDA | | USP10 | | | SEMG1 | | | GNG3 | | IGKV2-30 | | OSBPL10 | |  | | | CYP27B1 | | LAG3 | | RCAN3 | | TRBC2 | |  | | |  |
| KLF1 |  | | CXCR1 | | MPZL1 | | VMP1 | | | SIGLEC8 | | | GPM6A | | IGKV2-40 | | P2RX5 | |  | | | DCP1B | | LAIR2 | | REG4 | | TRBJ1-2 | |  | | |  |
| L1TD1 |  | | CXCR2 | | MSL1 | | VNN2 | | | SLC16A14 | | | GPR17 | | IGKV2D-24 | | PAX5 | |  | | | DGKA | | LAMA2 | | RGCC | | TRBV12-3 | |  | | |  |
| LILRA1 |  | | CYP4F3 | | MX2 | | VPS9D1 | | | SLC29A1 | | | GPRC5D | | IGKV2D-26 | | PCDHGA12 | |  | | | DKK3 | | LEF1 | | RGPD2 | | TRBV12-4 | |  | | |  |
| LKAAEAR1 |  | | CYSTM1 | | MXD1 | | WDFY3 | | | SMIM1 | | | GRAPL | | IGKV2D-28 | | PKIG | |  | | | DLGAP5 | | LGR6 | | RHOXF1 | | TRBV14 | |  | | |  |
| LPL |  | | DEFA1 | | N4BP1 | | XPO6 | | | SVOPL | | | H3C7 | | IGKV2D-29 | | PLAAT2 | |  | | | DPP4 | | LIM2 | | ROR2 | | TRBV19 | |  | | |  |
| LYNX1 |  | | DEFA3 | | NAMPT | | ZDHHC18 | | | TFF3 | | | H4C4 | | IGKV2D-30 | | PLEKHG1 | |  | | | DPY19L2 | | LMCD1 | | RORA | | TRBV2 | |  | | |  |
| LYPD2 |  | | DEFA4 | | NBEAL2 | | ZFP36L1 | | | THBS4 | | | HLA-DOB | | IGKV2D-40 | | PLEKHG7 | |  | | | DSC1 | | LNP1 | | RORC | | TRBV20-1 | |  | | |  |
| MAFB |  | | DHRS13 | | NECAB2 | | ZNF467 | | | TMEM273 | | | IGF1 | | IGKV3-11 | | PLPP3 | |  | | | DTHD1 | | LONRF3 | | RTKN2 | | TRBV23-1 | |  | | |  |
| MARCO |  | | DHX34 | | NPR3 | | ZNF658 | | | TMIGD3 | | | IGHA1 | | IGKV3-15 | | POF1B | |  | | | DTL | | LOXL1 | | RTN4R | | TRBV27 | |  | | |  |
| MERTK |  | | DRC1 | | NRBF2 | |  | | | TNNT1 | | | IGHA2 | | IGKV3-20 | | POU2AF1 | |  | | | DUSP16 | | LRRN3 | | S100B | | TRBV28 | |  | | |  |
| MRAS |  | | DUSP1 | | NSUN7 | |  | | | TRPC6 | | | IGHG1 | | IGKV3-7 | | PTPRK | |  | | | DUSP4 | | MAL | | S1PR5 | | TRBV3-1 | |  | | |  |
| MS4A14 |  | | DUSP13 | | NTNG2 | |  | | | TWIST2 | | | IGHG2 | | IGKV3D-15 | | QRSL1 | |  | | | ECRG4 | | MAN1C1 | | SARDH | | TRBV4-2 | |  | | |  |
| MS4A7 |  | | DYNLT4 | | ORM1 | |  | | | VLDLR | | | IGHG4 | | IGKV3D-20 | | RAB30 | |  | | | EDAR | | MATN2 | | SEC14L2 | | TRBV5-1 | |  | | |  |
| MSR1 |  | | DYSF | | ORM2 | |  | | | VSTM1 | | | IGHM | | IGKV3D-7 | | RALGPS2 | |  | | | EFNA1 | | MB21D2 | | SEMA3G | | TRBV5-3 | |  | | |  |
| MYO10 |  | | EGLN1 | | OTX1 | |  | | | ZBTB42 | | | IGHV1-18 | | IGKV4-1 | | RASSF6 | |  | | | ELOVL4 | | MCF2L2 | | SFRP5 | | TRBV5-7 | |  | | |  |
| MYO7A |  | | ELAPOR1 | | PF4 | |  | | |  | | | IGHV1-2 | | IGKV5-2 | | SCN3A | |  | | | ENPP5 | | ME1 | | SIRPG | | TRBV6-2 | |  | | |  |
| NAAA |  | | ERVH48-1 | | PGM5 | |  | | |  | | | IGHV1-24 | | IGKV6-21 | | SDR16C5 | |  | | | ENSG00000249209 | | MELK | | SLC1A7 | | TRBV6-4 | |  | | |  |
| NEFH |  | | FAM174A | | PHC2 | |  | | |  | | | IGHV1-3 | | IGKV6D-41 | | SLC16A4 | |  | | | EPHA1 | | MEOX1 | | SLC35G2 | | TRBV6-5 | |  | | |  |

**Supplementary Table 4: Clinical characteristics of patients with COPD.** Median (IQR) for all 18 included COPD patients.

| **Characteristic** | **N = 18** |
| --- | --- |
| Pack years, years | 43 (35, 65) |
| AECOPD preceeding year, n | 2.50 (1.25, 3.00) |
| Stable, n | 15 (83%) |
| Current smoker, n | 0 (0%) |
| α-1 antitrypsin deficiency, n | 0 (0%) |
| RV, L | 4.98 (4.14, 5.46) |
| RVpp, % | 208.5 (191.6, 250.5) |
| FEV_1_, L | 0.87 (0.73, 1.16) |
| FEV_1_pp, % | 32.80 (28.45, 37.57) |
| MEF_25_, L/min | 0.20 (0.15, 0.23) |
| MEF_25_pp, % | 31.50 (21.80, 43.44) |
| MEF_50_, L/min | 0.38 (0.30, 0.44) |
| MEF_50_pp, % | 15.55 (13.83, 19.90) |
| FEV_1_/FVC ratio, AU | 40.60 (37.97, 46.11) |
| ICS, n | 12 (67%) |
| AB p.o. | 5 (28%) |
| AB i.v., n | 5 (28%) |
| AECOPD, n | 2 (11%) |
| Pneumonia, n | 2 (11%) |
| Steroid p.o., n | 4 (22%) |
| Valves in situ, n | 5 (28%) |
| Condition after ELVR, n | 7 (39%) |
| Condition after LVRS, n | 1 (5.6%) |
| Pulmonary emphysema, n | 17 (94.4%) |

Abbreviations: AECOPD: acute exacerbations of chronic obstructive pulmonary disease; RV: residual volume; RVpp: residual volume, percent predicted; FEV_1_: forced expiratory volume in one second; FEV_1_pp: forced expiratory volume in one second, percent predicted; MEF_25_: maximal expiratory flow at 25% of forced vital capacity; MEF_25_pp: maximal expiratory flow at 25% of forced vital capacity, percent predicted; MEF_50_: maximal expiratory flow at 50% of forced vital capacity; MEF_50_pp: maximal expiratory flow at 50% of forced vital capacity, percent predicted; FEV_1_/FVC: ratio of forced expiratory volume in one second to forced vital capacity; AU: arbitrary units; ICS: inhaled corticosteroids; AB p.o.: oral antibiotics; AB i.v.: intravenous antibiotics; Steroid p.o.: oral corticosteroids; ELVR: endoscopic lung volume reduction; LVRS: lung volume reduction surgery.

**References:**

1. Taggart C*, et al.* Protean proteases: at the cutting edge of lung diseases. *Eur Respir J* **49**, (2017).

2. Chalmers JD*, et al.* Neutrophil Elastase Activity Is Associated with Exacerbations and Lung Function Decline in Bronchiectasis. *Am J Respir Crit Care Med* **195**, 1384-1393 (2017).

3. Mayer-Hamblett N*, et al.* Association between pulmonary function and sputum biomarkers in cystic fibrosis. *Am J Respir Crit Care Med* **175**, 822-828 (2007).

4. Hagner M*, et al.* New method for rapid and dynamic quantification of elastase activity on sputum neutrophils from patients with cystic fibrosis using flow cytometry. *European Respiratory Journal* **55**, (2020).

5. Callahan BJ, McMurdie PJ, Rosen MJ, Han AW, Johnson AJ, Holmes SP. DADA2: High-resolution sample inference from Illumina amplicon data. *Nat Methods* **13**, 581-583 (2016).

6. Müller T, Kalxdorf M, Longuespée R, Kazdal DN, Stenzinger A, Krijgsveld J. Automated sample preparation with SP 3 for low‐input clinical proteomics. *Molecular Systems Biology* **16**, e9111 (2020).

7. Heming S*, et al.* MSPypeline: A python package for streamlined data analysis of mass spectrometry-based proteomics. *Bioinformatics Advances* **2**, vbac004 (2022).

8. Vizcaino JA*, et al.* The PRoteomics IDEntifications (PRIDE) database and associated tools: status in 2013. *Nucleic Acids Res* **41**, D1063-1069 (2013).

9. Huber W, von Heydebreck A, Sultmann H, Poustka A, Vingron M. Variance stabilization applied to microarray data calibration and to the quantification of differential expression. *Bioinformatics* **18 Suppl 1**, S96-104 (2002).

10. McMurdie PJ, Holmes S. phyloseq: An R Package for Reproducible Interactive Analysis and Graphics of Microbiome Census Data. *PLOS ONE* **8**, e61217 (2013).

11. Love MI, Huber W, Anders S. Moderated estimation of fold change and dispersion for RNA-seq data with DESeq2. *Genome Biology* **15**, 550 (2014).

12. Argelaguet R*, et al.* MOFA+: a statistical framework for comprehensive integration of multi-modal single-cell data. *Genome Biology* **21**, 111 (2020).

13. Tibshirani R, Walther G, Hastie T. Estimating the Number of Clusters in a Data Set Via the Gap Statistic. *Journal of the Royal Statistical Society Series B: Statistical Methodology* **63**, 411-423 (2001).
